# Supplementary material for: The coefficient of determination R2 and intra-class correlation coefficient from generalized linear mixed-effects models revisited and expanded
Source: J R Soc Interface. 2017 Sep 13;14(134):20170213. doi: 10.1098/rsif.2017.0213 (PMC5636267; doi:10.1098/rsif.2017.0213)
Supplement: Appendices S1-S5 and S7 [file rsif20170213supp1.pdf]

Appendices S1-S5 and S7 for "The coefficient of determination R^2 and intra-class correlation ICC from generalized linear-mixed effects models revisited and expanded"

Shinichi Nakagawa
University of New South Wales, Sydney, Australia
Paul C. D. Johnson
University of Glasgow, UK
Holger Schielzeth
Friedrich Schiller University Jena, Germany

06 July 2017

Appendix S1

Deriving the observation-level variance σ_ε^2 using log-normal approximation for the negative binomial distribution with log link

Here, we only deal with the case of the negative binomial distribution, but this derivation process is directly applicable to the quasi-Poisson and gamma distributions with log link. Given a random variable x is negative binomially distributed, the mean and variance of x are respectively:

$$E[x] = \lambda,$$
$$\mathrm{var}[x] = \lambda + \frac{\lambda^2}{\theta},$$

where λ and θ are defined as in Table 1. When the distribution of $\ln(x)$ follows the natural logarithm of a log-normal distribution. Then, the variance of $\ln(x)$ is:

$$\mathrm{var}[\ln(x)] = \ln\left(1 + \frac{\mathrm{var}[x]}{E[x]^2}\right).$$

Therefore:

$$\mathrm{var}[\ln(x)] = \ln\left(1 + \frac{\lambda + \lambda^2/\theta}{\lambda^2}\right).$$

By rearranging, we obtain:

$$\mathrm{var}[\ln(x)] = \ln\left(1 + \frac{1}{\lambda} + \frac{1}{\theta}\right).$$

which is the observation-level variance for the negative binomial distribution with the log link function derived using the log-normal approximation.

Appendix S2

Comparison of the three methods for obtaining the observation-level variance σ_ε^2 for the Poisson distribution

We plot three different methods for obtaining the observation-level variance (formally we refered this as the distribution-specific variance for Poisson; for details see Appendix S4). We first load the packages that are need in the calculations:

```
# install.packages('latex2exp') # install it if you do not have this
library(latex2exp)  # enable to use LaTex in R expression
# install.packages('extremevalues') library(extremevalues) # this is needed
# unless running a commented-out part of the script
# install.packages('numDeriv')
library(numDeriv)  # we need a numerical method for getting derivatives of probit
```

Make sure you have installed and loaded all these packages to your current `R` session.

```
lnX <- seq(-20, 3, by = 0.001)
X <- exp(lnX)
plot(X, 1/X, type = "l", lty = "dotted", ylab = "Observation-level variance",
    xlab = TeX("$\\lambda$"), ylim = c(0, 10))
lines(X, log(1 + 1/X))
lines(X, trigamma(X), lty = "dashed")
legend(15, 10, c(TeX("$\\frac{1}{\\lambda}$"), TeX("$\\ln\\left(1+\\frac{1}{\\lambda}\\right)$"),
    TeX("$\\psi_1(\\lambda)$")), lty = c(3, 1, 2), bty = "n")
```

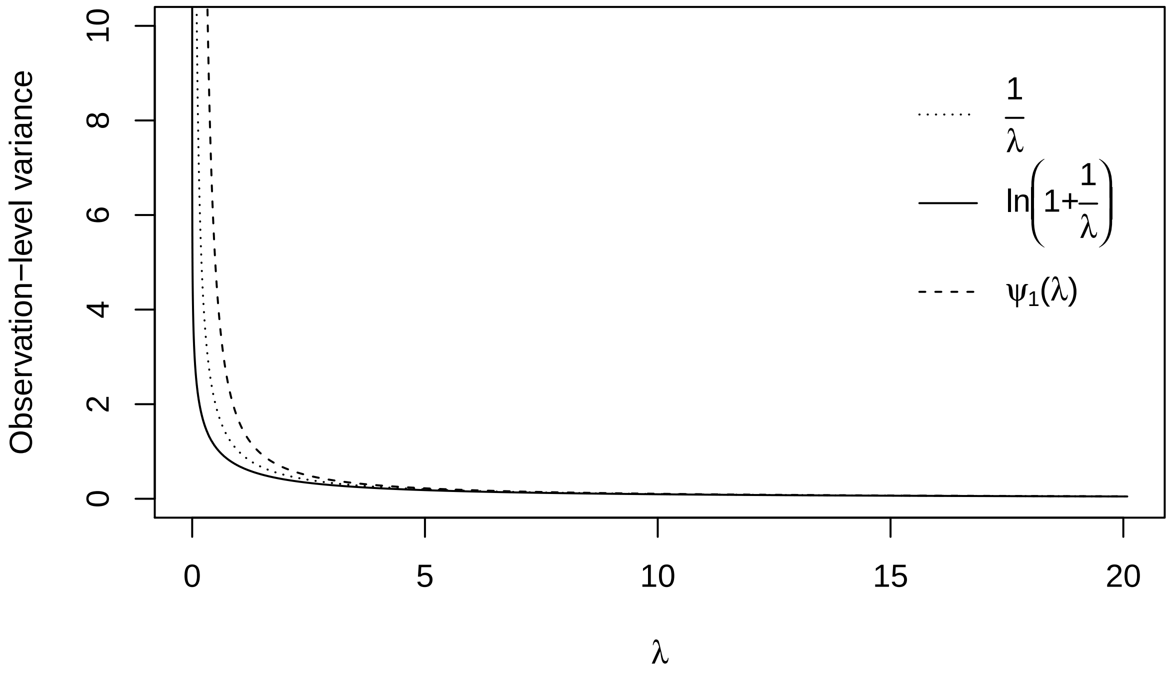

Figure 1: A comparsion of the three observation-level variance functions.

As you see, these three functions seem to converge for values larger than about 2 (Figure S1). Now we zoom into this figure.

```r
plot(X, 1/X, type = "l", lty = "dotted", ylab = "Observation-level variance",
    xlab = TeX("$\\lambda$"), ylim = c(0, 10), xlim = c(0, 3))
lines(X, log(1 + 1/X))
lines(X, trigamma(X), lty = "dashed")
legend(2, 10, c(TeX("$\\frac{1}{\\lambda}$"), TeX("$\\ln\\left(1+\\frac{1}{\\lambda}\\right)$"),
    TeX("$\\psi_1(\\lambda)$")), lty = c(3, 1, 2), bty = "n")
```

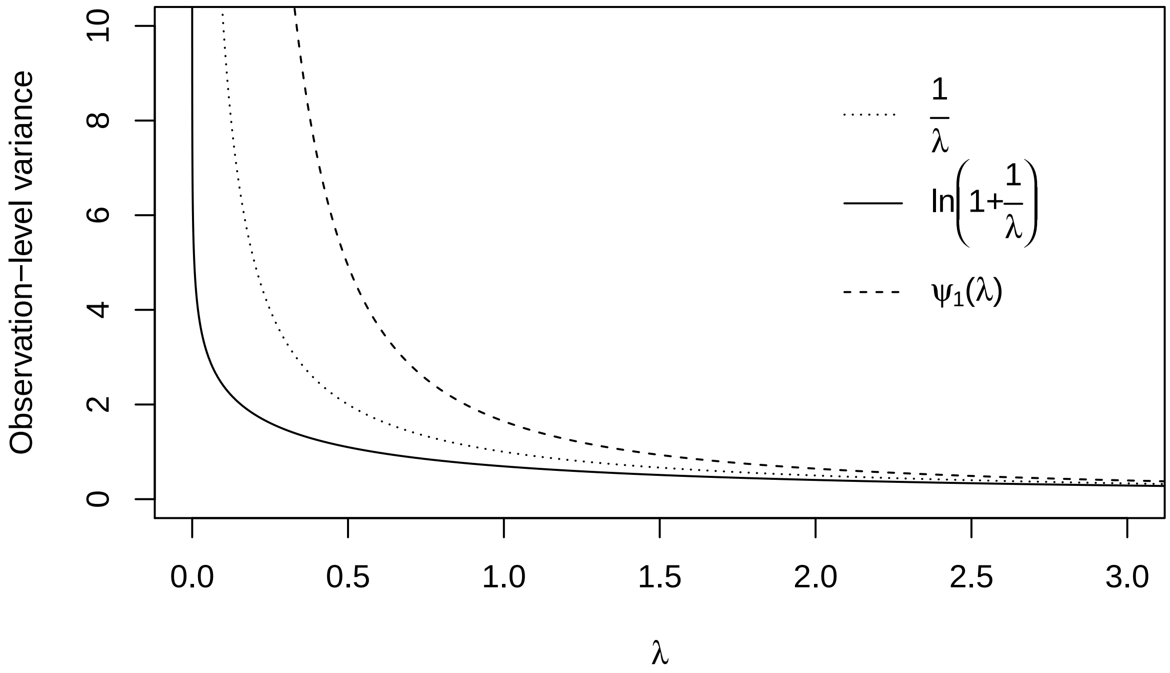

Figure 2: A comparsion of the three observation-level variance functions zoomed in.

We see substantial divergence among the three functions at small values of λ (Figure S2). Therefore, it is important to report which method is used when calculating R^2_{GLMM} and ICC_{GLMM}, especially with small λ.

Incidentally, we can obtain the delta method version of σ^2_ε using the R function `D`, without having to do derivations by hand!

```r
FunX <- expression(log(X))  # function of X
DXFunX <- D(FunX, "X")  # getting a derivative with respect to X
DXFunX  # this is 1/lambda and lambda*(1/lambda)^2 will be 1/lambda
```

```
## 1/X
```

```r
# the delta method for variance approximation (Delta 1)
VarOd <- X * eval(DXFunX)^2
plot(X, 1/X, type = "l", lty = "dotted", ylab = "Observation-level variance",
    xlab = TeX("$\\lambda$"), lwd = 4, ylim = c(0, 10), xlim = c(0, 3))
points(X, VarOd, type = "l", lty = "dashed", col = "red", lwd = 2)
legend(1.5, 10, c(TeX("$\\frac{1}{\\lambda}$"), "", "D function in R"), lty = c(3,
    0, 2), col = c("black", "black", "red"), bty = "n", )
```

There is an exact match between the results from the delta method and the delta method outcome `VarOd` as both are $\frac{1}{\lambda}$ (`1/X`).

It is also very important to realize that when $\frac{1}{\lambda}$ (Poisson distributions), $\frac{1}{\lambda}+\frac{1}{\theta}$ (negative-binomial distributions) or $\frac{1}{\nu}$ (gamma distributions) are under 0.5, estimates of the observation-level variance σ^2_ε from the three

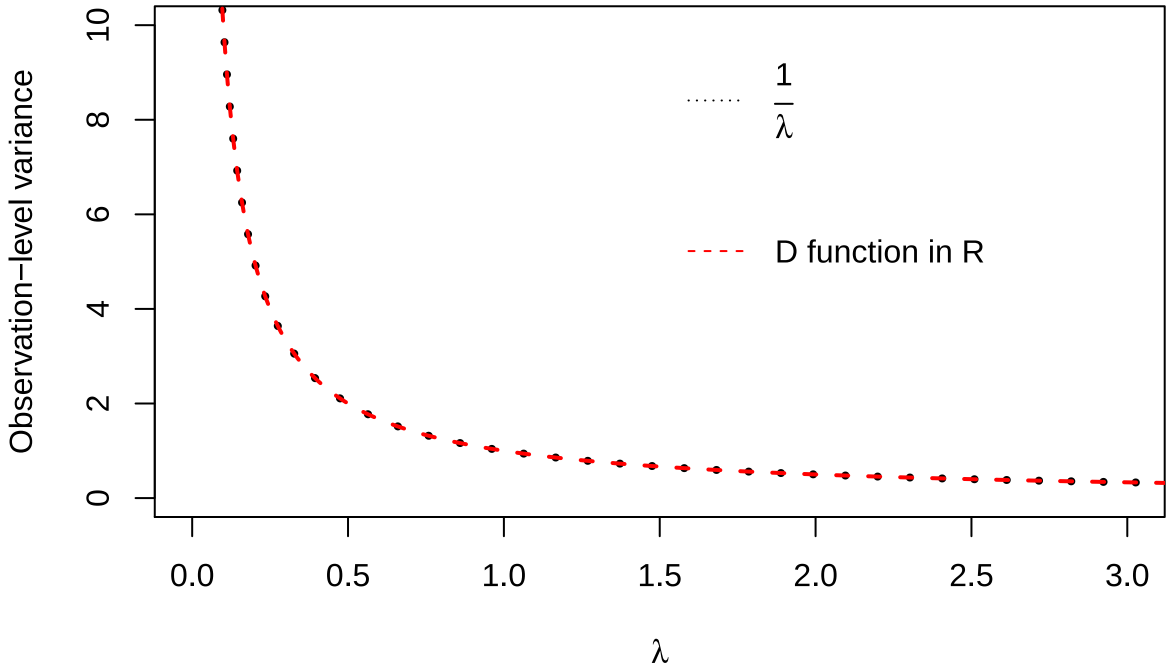

Figure 3: A comparsion of alternative approaches for applying the delta method.

methods can be noticeable different. This can also be seen in the worked examples (Appendix S6). Our recommendation is to use the traigamma function approach, which we did in our worked examples.

Appendix S3

Looking into the performance of the delta method for bias corrections

Below we compare the exact mean (equation (5.8)) and the approximated mean (equation (6.2)) under 3 different variance values ($\sigma^2_\tau = 0.25$, 0.5 and 1) with Poisson (count) data.

```
Beta <- seq(-4, 4, by = 0.05)
VarQuarter <- 0.25
VarHalf <- 0.5
VarOne <- 1

FunB1 <- expression(exp(Beta))  # inverse of log or exp
DBFunB1 <- D(FunB1, "Beta")  # taking derivative of FunB1

lnExactQuarter <- exp(Beta + 0.5 * VarQuarter)
lnApproxQuarter <- exp(Beta) + 0.5 * VarQuarter * eval(DBFunB1)
lnExactHalf <- exp(Beta + 0.5 * VarHalf)
lnApproxHalf <- exp(Beta) + 0.5 * VarHalf * eval(DBFunB1)
lnExactOne <- exp(Beta + 0.5 * VarOne)
lnApproxOne <- exp(Beta) + 0.5 * VarOne * eval(DBFunB1)

plot(lnExactQuarter, lnApproxQuarter, type = "l", ylab = "Approximated mean by the delta method",
    xlab = "Exact mean", xlim = c(0, 20), ylim = c(0, 20))
lines(lnExactHalf, lnApproxHalf, lty = 2)
```

```
lines(lnExactOne, lnApproxOne, lty = 3)
abline(0, 1, col = "red")
legend(0, 20, c(TeX("$\\sigma^2_{\\tau} = 0.25"), TeX("$\\sigma^2_{\\tau} = 0.5"),
    TeX("$\\sigma^2_{\\tau} = 1")), lty = c(1, 2, 3), bty = "n")
```

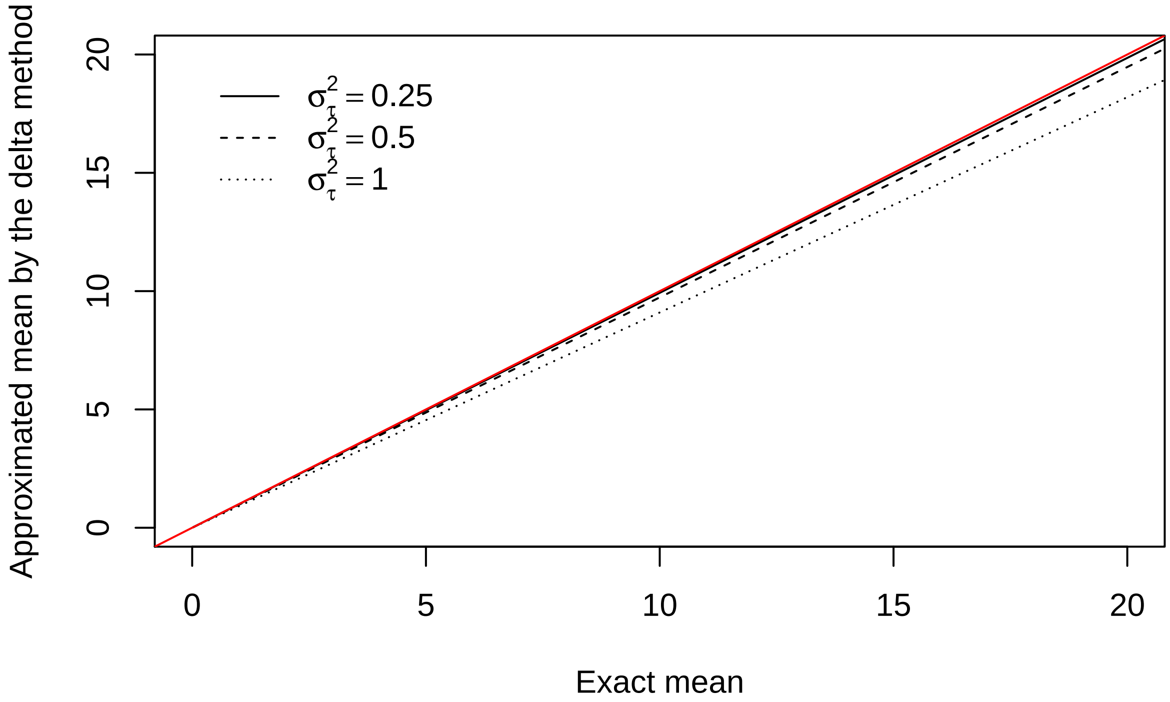

Figure 4: Performance of approximations (black) against unbiased line for Poission (count) data with the log-link.

As is visibale from Figure S4, the delta method approximation starts to perform worse with larger mean values and also larger variance values.

Now we look at the performance of three approximations of mean values (equations (6.7)-(6.9)); we will call equation (6.7) the delta approximation, equation (6.7) the tanh (hyperbolic tangent) approximaiton, equation (6.8) the normal approximation (because this approximation uses the similarity between the logistic distribution and the normal distribution (see equation (6.10)). Note that in this case (proportion data with the logit link), we have to use simulations to obtain correct mean values.

```
Beta <- seq(-10, 10, by = 0.05)
FunB2 <- expression(exp(Beta)/(1 + exp(Beta)))
DBDBFunB2 <- D(D(FunB2, "Beta"), "Beta")  # taking derivative of FunB2 twice

# getting unbiased means using simulations
logitSimQuarter <- logitSimHalf <- logitSimOne <- 1:length(Beta)
for (i in 1:length(Beta)) {
    logitSimQuarter[i] <- mean(plogis(Beta[i] + rnorm(1e+06, 0, sqrt(VarQuarter))))
    logitSimHalf[i] <- mean(plogis(Beta[i] + rnorm(1e+06, 0, sqrt(VarHalf))))
    logitSimOne[i] <- mean(plogis(Beta[i] + rnorm(1e+06, 0, sqrt(VarOne))))
}

logitApprox1Quarter <- eval(FunB2) + 0.5 * VarQuarter * eval(DBDBFunB2)
logitApprox2Quarter <- plogis(Beta - 0.5 * VarQuarter * tanh(Beta * (1 + 2 *
```

```r
    exp(-0.5 * VarQuarter))/6))
logitApprox3Quarter <- plogis(Beta/sqrt(1 + ((16 * sqrt(3))/(15 * pi))^2 * VarQuarter))
logitApprox1Half <- eval(FunB2) + 0.5 * VarHalf * eval(DBDBFunB2)
logitApprox2Half <- plogis(Beta - 0.5 * VarHalf * tanh(Beta * (1 + 2 * exp(-0.5 *
    VarHalf))/6))
logitApprox3Half <- plogis(Beta/sqrt(1 + ((16 * sqrt(3))/(15 * pi))^2 * VarHalf))
logitApprox1One <- eval(FunB2) + 0.5 * VarOne * eval(DBDBFunB2)
logitApprox2One <- plogis(Beta - 0.5 * VarOne * tanh(Beta * (1 + 2 * exp(-0.5 *
    VarOne))/6))
logitApprox3One <- plogis(Beta/sqrt(1 + ((16 * sqrt(3))/(15 * pi))^2 * VarOne))

plot(logitSimQuarter, logitApprox1Quarter, type = "l", ylab = "Approximated mean by the two methods",
    xlab = "Simulated mean (unbiased)")
lines(logitSimHalf, logitApprox1Half, lty = 2)
lines(logitSimOne, logitApprox1One, lty = 3)
lines(logitSimQuarter, logitApprox2Quarter, lty = 1, col = "green")
lines(logitSimHalf, logitApprox2Half, lty = 2, col = "green")
lines(logitSimOne, logitApprox2One, lty = 3, col = "green")
lines(logitSimQuarter, logitApprox3Quarter, lty = 1, col = "blue")
lines(logitSimHalf, logitApprox3Half, lty = 2, col = "blue")
lines(logitSimOne, logitApprox3One, lty = 3, col = "blue")
abline(0, 1, col = "red")
legend(0, 1, c(TeX("$\\sigma^2_{\\tau} = 0.25 (delta)"), TeX("$\\sigma^2_{\\tau} = 0.5 (delta)"),
    TeX("$\\sigma^2_{\\tau} = 1 (delta)"), TeX("$\\sigma^2_{\\tau} = 0.25 (tanh)"),
    TeX("$\\sigma^2_{\\tau} = 0.5 (tanh)"), TeX("$\\sigma^2_{\\tau} = 1 (tanh)"),
    TeX("$\\sigma^2_{\\tau} = 0.25 (normal)"), TeX("$\\sigma^2_{\\tau} = 0.5 (normal)"),
    TeX("$\\sigma^2_{\\tau} = 1 (normal)")), lty = c(1, 2, 3, 1, 2, 3, 1, 2,
    3), co = c(rep(c("black", "green", "blue"), each = 3)), bty = "n", cex = 0.7)
```

From Figure S5 this is hard to see differences between the two methods so we zoom in apart from deviations occur most at around 0.69 an 0.71.

```r
plot(logitSimQuarter, logitApprox1Quarter, type = "l", ylab = "Approximated mean by the two methods",
    xlab = "Simulated mean (unbiased)", xlim = c(0.69, 0.71), ylim = c(0.69,
        0.71))
lines(logitSimHalf, logitApprox1Half, lty = 2)
lines(logitSimOne, logitApprox1One, lty = 3)
lines(logitSimQuarter, logitApprox2Quarter, lty = 1, col = "green")
lines(logitSimHalf, logitApprox2Half, lty = 2, col = "green")
lines(logitSimOne, logitApprox2One, lty = 3, col = "green")
lines(logitSimQuarter, logitApprox3Quarter, lty = 1, col = "blue")
lines(logitSimHalf, logitApprox3Half, lty = 2, col = "blue")
lines(logitSimOne, logitApprox3One, lty = 3, col = "blue")
abline(0, 1, col = "red")
legend(0.69, 0.71, c(TeX("$\\sigma^2_{\\tau} = 0.25 (delta)"), TeX("$\\sigma^2_{\\tau} = 0.5 (delta)"),
    TeX("$\\sigma^2_{\\tau} = 1 (delta)"), TeX("$\\sigma^2_{\\tau} = 0.25 (tanh)"),
    TeX("$\\sigma^2_{\\tau} = 0.5 (tanh)"), TeX("$\\sigma^2_{\\tau} = 1 (tanh)"),
    TeX("$\\sigma^2_{\\tau} = 0.25 (normal)"), TeX("$\\sigma^2_{\\tau} = 0.5 (normal)"),
    TeX("$\\sigma^2_{\\tau} = 1 (normal)")), lty = c(1, 2, 3, 1, 2, 3, 1, 2,
    3), co = c(rep(c("black", "green", "blue"), each = 3)), bty = "n", cex = 0.7)
```

It seems all approximation do worse as σ^2_τ increases. As one can see the tanh approximation performs best.

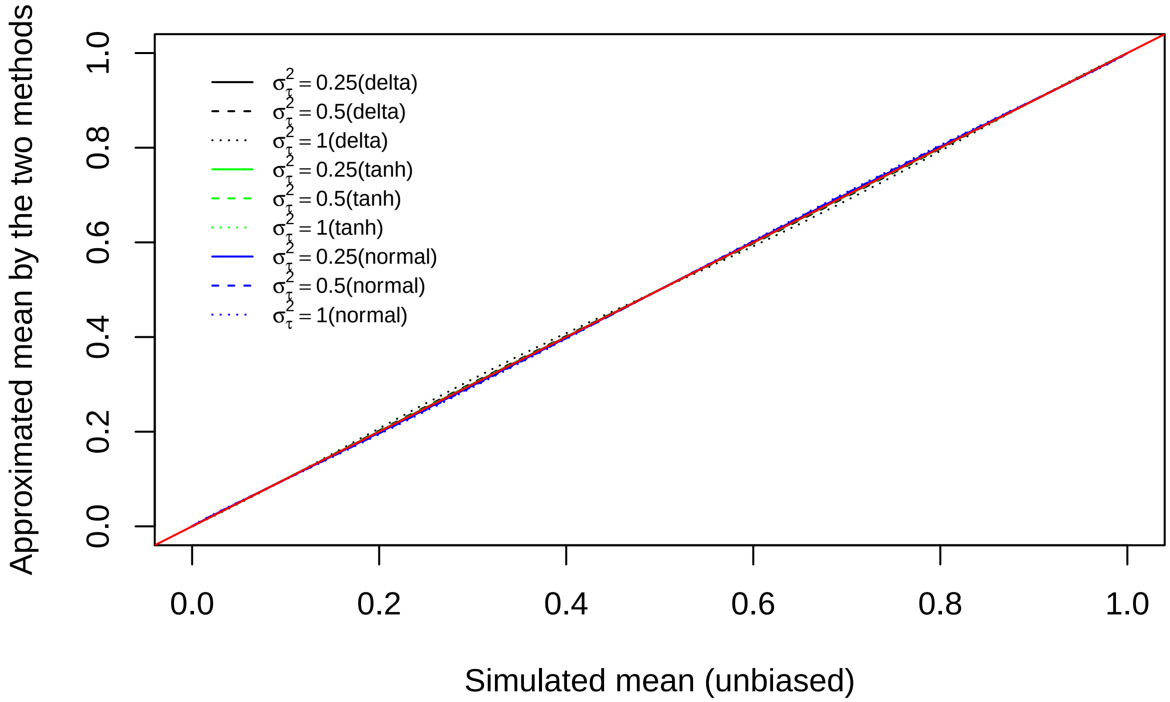

Figure 5: Performance of approximations (black) against unbiased line for binomial data with the logit-link (red).

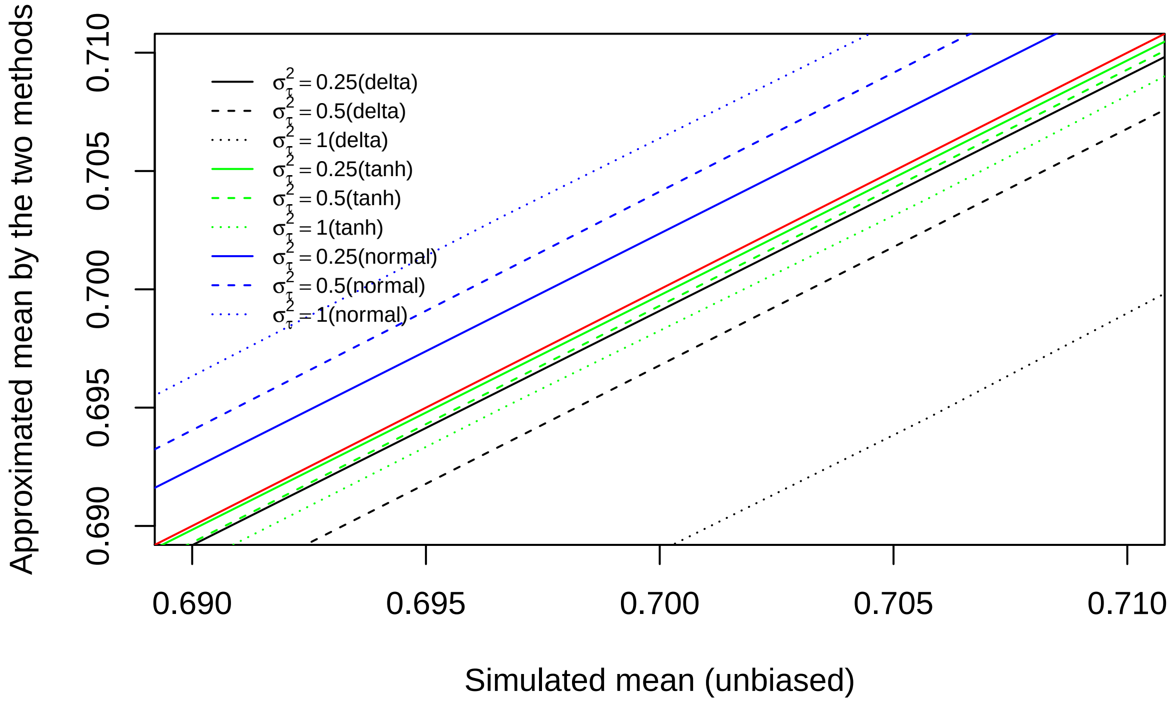

Figure 6: Zooming in on the performance of approximations (black) against unbiased line for binomial data with the logit-link (red).

Appendix S4

Why R^2_{GLMM} and ICC_{GLMM} using variances on the latent scale are estiamted on the data/original scale

Here, we use R^2_{GLMM} and ICC_{GLMM} as calculated using the 'delta-method-based' observation-level variance.

Marginal R^2_{GLMM} from a quasi-Poisson GLMM (model 2 in the main text) using the variance components and the obervation-level variance (note both are on the latent scale) can be expressed as:

$$R^2_{\text{QP}-\ln(m)} = \frac{\sigma^2_f}{\sigma^2_f + \sigma^2_\alpha + \sigma^2_\epsilon}.$$

By applying the delta method for variance approximation, we can approximate R^2_{GLMM} on the data/orignal scale can be written as:

$$R^2_{\text{QP}-\ln(m)*} \approx \frac{\sigma^2_f \left(\frac{dg(\beta_0)}{d\beta_0}\right)^2}{\left(\sigma^2_f + \sigma^2_\alpha + \sigma^2_\epsilon\right)\left(\frac{dg(\beta_0)}{d\beta_0}\right)^2},$$

where g is the transformation function (inverse link function).

By simplifying this, we obtain:

$$R^2_{\text{QP}-\ln(m)*} \approx \frac{\sigma^2_f}{\sigma^2_f + \sigma^2_\alpha + \sigma^2_\epsilon} = R^2_{\text{QP}-\ln(m)}.$$

This argument above is directly transferable to ICC_{GLMM} and to other non-Gaussian distributions. Thus, R^2_{GLMM} and ICC_{GLMM} using variances on the latent scale approximates to R^2_{GLMM} and ICC_{GLMM} on data/orignal scale. Also, this implies that ICC on the data/orignal scale can be written by using the binomial (binary) GLMM when $n = 1$ (Model 6):

$$\text{ICC}_{\text{binom}-\text{logit}*} \approx \frac{\sigma^2_\alpha p^2/(1+e^b)^2}{(\sigma^2_\alpha + \sigma^2_e)p^2/(1+e^b)^2 + p(1-p)},$$

where p is the mean on the data scale and b is the corresponding value on the latent scale and $p = e^b/(1+e^b)$; this was first derived in Browne et al. (2005, *J. R. Statstic. Soc. A.*, 168: 599-613) using the delta method. An ICC can be approximated by using the delta method and then, the observation-level σ^2_ϵ for the binomial distriubtion with the logit link (based on the delta method) is $1/p(1-p)$ when $n = 1$ (see Table 2):

$$\text{ICC}_{\text{binom}-\text{logit}} \approx \frac{\sigma^2_\alpha}{\sigma^2_\alpha + \sigma^2_e + 1/p(1-p)}.$$

Given $p = e^b/(1+e^b)$, $p(1-p) = e^b/(1+e^b)^2$ and also $e^b = p/(1-p)$ and therefore, $(1+e^b)^2 = 1/(1-p)^2$. It follows that ICC on the data scale can be re-written as:

$$\text{ICC}_{\text{binom}-\text{logit}*} \approx \frac{\sigma^2_\alpha p^2(1-p)^2}{(\sigma^2_\alpha + \sigma^2_e)p^2(1-p)^2 + p(1-p)},$$

By dividing both the numerator and denominator by $p^2(1-p)^2$, we have:

$$\text{ICC}_{\text{binom-logit}*} \approx \frac{\sigma_\alpha^2}{\sigma_\alpha^2 + \sigma_e^2 + 1/p(1-p)}.$$

This is the same as the ICC formula above. Also, a more general formulat for $\text{ICC}_{\text{binom-logit}}$ is:

$$\text{ICC}_{\text{binom-logit}} \approx \frac{\sigma_\alpha^2}{\sigma_\alpha^2 + \sigma_e^2 + 1/np(1-p)}.$$

Appendix S5

Comparing the distribution-specific and observation-level variance for the three common link functions of the binomial distribution

We plot how the 'delta-method-based' observation-level variance (when $n = 1$) change as p (probability; `Prob`) changes for the logit, probit and complementary log-log link function along with the corresponding 'theortical' distribution-specific variance.

```
Prob <- seq(1e-04, 0.9999, by = 1e-04)
FunPlogit <- expression(log(Prob/(1 - Prob)))  # logit
FunPcclog <- expression(log(-log(1 - Prob)))  # c-c log

DPFunPlogit <- D(FunPlogit, "Prob")  # derivative of logit
DPFunPcclog <- D(FunPcclog, "Prob")  # derivative of cclog
# the delta method for variance approximation
VarOlogit <- Prob * (1 - Prob) * eval(DPFunPlogit)^2
# VarDlogit<-1/(Prob*(1-Prob)) # as in Table 2 - equivalent as above the
# delta method (note some differences from the others)
VarOprobit <- Prob * (1 - Prob) * grad(qnorm, Prob)^2
# VarDprobit<-2*pi*Prob*(1-Prob)*(exp((extremevalues::invErf(2*Prob-1))^2))^2
# as in Table 2 - equivalent as above the delta method
VarOcclog <- Prob * (1 - Prob) * eval(DPFunPcclog)^2
# VarDcclog<-Prob/((log(1-Prob))^2*(1-Prob)) # as in Table 2 - equivalent as
# above
```

Note that for the probit function, we had to use the numerical approach (`numDeriv package`) rather than the default `D` function. However, these functions listed in Table 3 can be directly used; they will produce the same results.

```
plot(Prob, VarOlogit, type = "l", ylab = "Variance", xlab = "Probability", ylim = c(0,
    20))
lines(Prob, VarOprobit, col = "red")
lines(Prob, VarOcclog, col = "blue")
abline(pi^2/3, 0, lty = "dashed")
abline(1, 0, lty = "dashed", col = "red")
abline(pi^2/6, 0, lty = "dashed", col = "blue")
legend(0.5, 20, c("Logit (link)", "Logit (latent)", "Probit (link)", "Probit (latent)",
    "CClog(link)", "CClog(latent)"), lty = c(1, 2, 1, 2, 1, 2), col = rep(c("black",
    "red", "blue"), each = 2), bty = "n")
```

It becomes clear form the corresponding figure that the observation-level variance is always larger than distribution-specific variance apart from the case of complementary-complementary (c-c) log link. It may not be surprising to see the observation-level variances increase at both extreme (0 and 1) because the

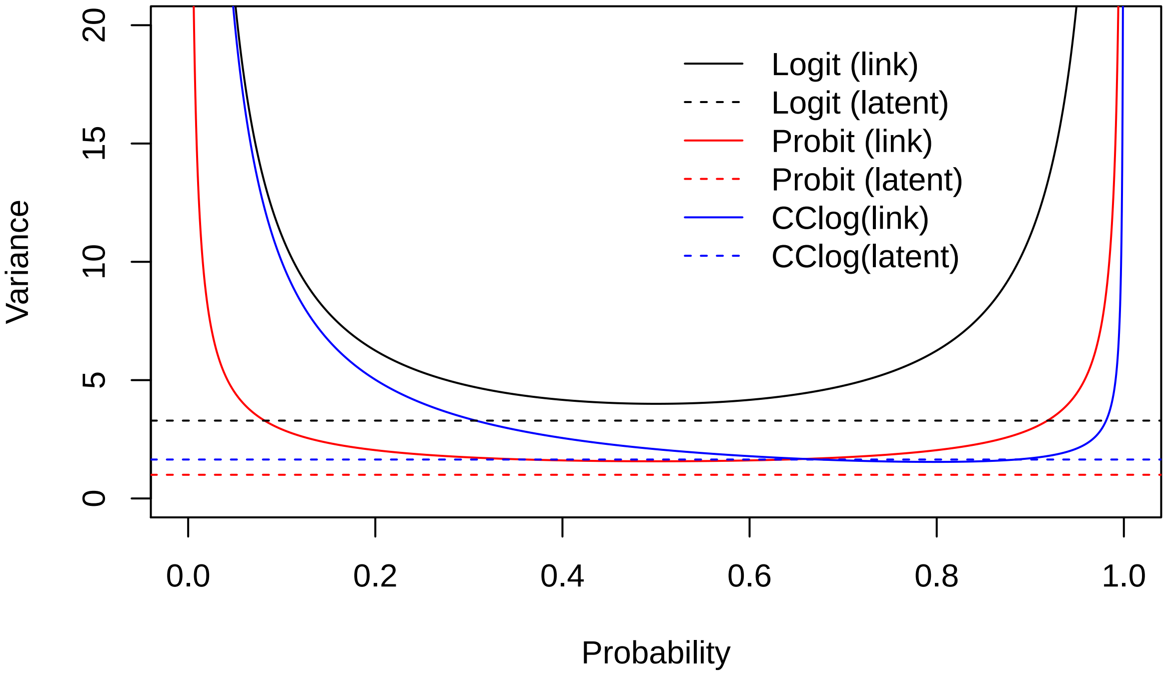

Figure 7: A comparision of distribution-specific and observation-level variances for the 3 common link functions.

total variance decreases and uncertainty increases near 0 and 1. It is hard to distinguish group-level form observation-level variance.

Appendix S7

R^2_{GLMM} and ICC_{GLMM} without σ^2_d

de Villemereuil and colleagues (2016, *Genetics*, 204: 1281-1294) demonstrated that hertaiblity on the latent scale can be caculated without σ^2_d. This is useful if the latent scale is the scale of interest, because selection can also be inferred on the latent scale. More broadly, this is difficult to apply to cases like binary data, because σ^2_e (overdisperson variance) is theortically zero and since σ^2_d is excluded from the calculateion of $\sigma^2_\epsilon = \sigma^2_e = 0$ (but see Browne et al., 2005, *J. R. Statstic. Soc. A.*, 168: 599-613). Consequently, de Villemereuil et al (2016) define R^2 and ICC for Models 5 & 6 usiing an additive overdisperson term to obtain σ^2_e:

$$R^2_{\text{GLMM}(m)}* = \frac{\sigma^2_f}{\sigma^2_f + \sigma^2_\alpha + \sigma^2_e}$$

$$R^2_{\text{GLMM}(c)}* = \frac{\sigma^2_f + \sigma^2_\alpha}{\sigma^2_f + \sigma^2_\alpha + \sigma^2_e}$$

$$\text{ICC}_{\text{GLMM}}* = \frac{\sigma^2_\alpha}{\sigma^2_\alpha + \sigma^2_e}$$

The main difference between R^2_{GLMM} and ICC_{GLMM}, and $R^2_{\text{GLMM}}*$ and $\text{ICC}_{\text{GLMM}}*$ are that $R^2_{\text{GLMM}}*$ and $\text{ICC}_{\text{GLMM}}*$ can be 1 while R^2_{GLMM} and ICC_{GLMM} can never become 1 (cf. Nagelkerke, 1991, *Biometrika*, 78: 691-692; this paper proposes how to make R^2 for generalized linear models, GLM have the maximum value of 1). $R^2_{\text{GLMM}}*$ and $\text{ICC}_{\text{GLMM}}*$ are likely to be useful when one sees the variance due to non-Guassian

distribution (distribution-specific variance) as some noise added on the data (original) scale. This may be analgous to taking out variance due to measurement error in the context of a Gaussian GLMM. We note that you can also add an overdisperson term to obtain σ_e^2 in Models 2-4 and calcuate $R^2_{\mathrm{GLMM}}*$ and $\mathrm{ICC}_{\mathrm{GLMM}}*$, which are distinct from R^2_{GLMM} and $\mathrm{ICC}_{\mathrm{GLMM}}$, which we defined in the main text.
