# Supplementary material for: The coefficient of determination R2 and intra-class correlation coefficient from generalized linear mixed-effects models revisited and expanded
Source: J R Soc Interface. 2017 Sep 13;14(134):20170213. doi: 10.1098/rsif.2017.0213 (PMC5636267; doi:10.1098/rsif.2017.0213)
Supplement: Worked examples (Appendix S6) [file rsif20170213supp2.pdf]

Appendix S6: Worked examples for "The coefficient of determination R^2 and intra-class correlation ICC from generalized linear-mixed effects models revisited and expanded"

Shinichi Nakagawa
University of New South Wales, Sydney, Australia
Paul C. D. Johnson
University of Glasgow, UK
Holger Schielzeth
Friedrich Schiller University Jena, Germany

06 July 2017

Contents

Data generation: revisting the beetles **2**
- Predictor variables . . . 2
- Response variables . . . 2
  - (i) Fecundity: the number of eggs per female . . . 2
  - (ii) Parasite: the number of endoparasite per individual . . . 3
  - (iii) Size: the body length (in mm) of an individual . . . 3
  - (iv) Exploraton: the time taken visiting five sectors (in sec) for an individual . . . 3
  - (v) Morph: Colour morph of a male . . . 4

Analysis **4**
- Preparation . . . 4
- (1) Fecudity models: Quasi-Poisson GLMMs with log link . . . 5
  - (1.1) `glmmadmb` fecundity models . . . 5
  - (1.2) `glmmPQL` fecundity models . . . 7
- (2) Parasite models: Negative binomial GLMMs with log link . . . 9
  - (2.1) `glmmadmb` parasite models . . . 9
  - (2.2) `glmer.nb` parasite models . . . 12
- (3) Size models: Gamma GLMMs with log link . . . 14
  - (3.1) `glmmadmb` size models . . . 14
  - (3.2) `glmmPQL` size models . . . 16
  - (3.3) `glmer` size models . . . 19
- (4) Exploration models: Gamma GLMMs with log link . . . 21
  - (4.1) `glmmadmb` exploration models . . . 21
  - (4.2) `glmPQL` exploration models . . . 23
  - (4.3) `glmer` exploration models . . . 26
- (5) Morph models: Binomial (binary) GLMMs with logit link . . . 28
  - (5.1) `glmmadmb` morph models . . . 28
  - (5.2) `glmer` morph models . . . 30
- (6) *Extra* parasite models: Tweedie (Compound Poisson) GLMMs with log link . . . 33
  - (6.1) `cpglmm` parasite models . . . 33

This is the end (Thank you) **35**

Data generation: revisting the beetles

Predictor variables

First, we start generating a data frame with all design variables.

```
# 12 different populations n = 960
Population <- gl(12, 80, 960)
# 120 containers (8 individuals in each container)
Container <- gl(120, 8, 960)
# Sex of the individuals. Uni-sex within each container (individuals are
# sorted at the pupa stage)
Sex <- factor(rep(rep(c("Female", "Male"), each = 8), 60))
# Habitat at the collection site: dry or wet soil (four indiviudal from each
# Habitat in each container)
Habitat <- factor(rep(rep(c("Dry", "Wet"), each = 4), 120))
# Food treatment at the larval stage: special food ('Exp') or standard food
# ('Cont')
Treatment <- factor(rep(c("Cont", "Exp"), 480))
# Data combined in a data frame
Data <- data.frame(Population = Population, Container = Container, Sex = Sex,
    Habitat = Habitat, Treatment = Treatment)
```

Response variables

(i) Fecundity: the number of eggs per female

```
# Subset the design matrix (only females lay eggs)
DataFemale <- Data[Data$Sex == "Female", ]
# set seed for reproduciblity (this will enable one to get the same data
# every time)
set.seed(777)
# simulation of the underlying random effects (Population and Container with
# variance of 0.4 and 0.05, respectively)
PopulationE <- rnorm(12, 0, sqrt(0.4))
ContainerE <- rnorm(120, 0, sqrt(0.05))
# generation of response values on latent scale (!) based on fixed effects,
# random effects and residual errors
EggL <- with(DataFemale, 1.1 + 0.5 * (as.numeric(Treatment) - 1) + 0.1 * (as.numeric(Habitat) -
    1) + PopulationE[Population] + ContainerE[Container] + rnorm(480, 0, sqrt(0.1)))
# data generation (on data scale!) based on Poisson distribution
DataFemale$Egg <- rpois(length(EggL), exp(EggL))
# looking at the data frame
str(DataFemale)
```

```
## 'data.frame':    480 obs. of  6 variables:
##  $ Population: Factor w/ 12 levels "1","2","3","4",..: 1 1 1 1 1 1 1 1 1 1 ...
##  $ Container : Factor w/ 120 levels "1","2","3","4",..: 1 1 1 1 1 1 1 1 3 3 ...
##  $ Sex       : Factor w/ 2 levels "Female","Male": 1 1 1 1 1 1 1 1 1 1 ...
##  $ Habitat   : Factor w/ 2 levels "Dry","Wet": 1 1 1 1 2 2 2 2 1 1 ...
##  $ Treatment : Factor w/ 2 levels "Cont","Exp": 1 2 1 2 1 2 1 2 1 2 ...
##  $ Egg       : int  4 5 3 0 4 11 13 3 7 18 ...
```

(ii) Parasite: the number of endoparasite per individual

```
# Data frame for both sex
DataAll <- Data
# simulation of the underlying random effects (Population and Container with
# variance of 0.5 and 0.8, respectively)
PopulationE <- rnorm(12, 0, sqrt(0.5))
ContainerE <- rnorm(120, 0, sqrt(0.8))
# generation of response values on latent scale (!) based on fixed effects
# and random effects
ParasiteL <- with(DataAll, 1.8 + 2 * (-1) * (as.numeric(Sex) - 1) + 0.8 * (-1) *
    (as.numeric(Treatment) - 1) + 0.7 * (as.numeric(Habitat) - 1) + PopulationE[Population] +
    ContainerE[Container])
# data generation (on data scale!) based on negative binomial distributions;
# size = theta
DataAll$Parasite <- rnbinom(length(ParasiteL), size = 5, mu = exp(ParasiteL))
```

(iii) Size: the body length (in mm) of an individual

```
# simulation of the underlying random effects (Population and Container with
# variance of 1.3 and 0.3, respectively)
PopulationE <- rnorm(12, 0, sqrt(1.3))
ContainerE <- rnorm(120, 0, sqrt(0.3))
# data generation based on fixed effects, random effects and random
# residuals errors
DataAll$BodyL <- 15 + 3 * (-1) * (as.numeric(Sex) - 1) + 0.4 * (as.numeric(Treatment) -
    1) + 0.15 * (as.numeric(Habitat) - 1) + PopulationE[Population] + ContainerE[Container] +
    rnorm(960, 0, sqrt(1.2))
```

(iv) Exploraton: the time taken visiting five sectors (in sec) for an individual

```
# simulation of the underlying random effects (Population and Container with
# variance of 0.2 and 0.2, respectively)
PopulationE <- rnorm(12, 0, sqrt(0.2))
ContainerE <- rnorm(120, 0, sqrt(0.2))
# generation of response values on latent scale (!) based on fixed effects
# and random effects
ExplorationL <- with(DataAll, 4 + 1 * (-1) * (as.numeric(Sex) - 1) + 2 * (as.numeric(Treatment) -
    1) + 0.5 * (-1) * (as.numeric(Habitat) - 1) + PopulationE[Population] +
    ContainerE[Container])
# data generation (on data scale!) based on gamma distribution; size = theta
DataAll$Exploration <- rgamma(length(ExplorationL), shape = exp(ExplorationL) *
    0.3, rate = 0.3)
# looking at the data farme
str(DataAll)
```

```
## 'data.frame':    960 obs. of  8 variables:
##  $ Population : Factor w/ 12 levels "1","2","3","4",..: 1 1 1 1 1 1 1 1 1 1 ...
##  $ Container  : Factor w/ 120 levels "1","2","3","4",..: 1 1 1 1 1 1 1 1 2 2 ...
##  $ Sex        : Factor w/ 2 levels "Female","Male": 1 1 1 1 1 1 1 1 2 2 ...
##  $ Habitat    : Factor w/ 2 levels "Dry","Wet": 1 1 1 1 2 2 2 2 1 1 ...
```

```
##  $ Treatment  : Factor w/ 2 levels "Cont","Exp": 1 2 1 2 1 2 1 2 1 2 ...
##  $ Parasite   : num  5 2 5 3 10 8 4 4 0 0 ...
##  $ BodyL      : num  17.3 16.9 15.4 16.1 14.3 ...
##  $ Exploration: num  80.7 350.8 32.2 397.1 33.9 ...
```

(v) Morph: Colour morph of a male

```
# Subset the design matrix (only males express colour morphs)
DataMale <- subset(Data, Sex == "Male")
# simulation of the underlying random effects (Population and Container with
# variance of 1.2 and 0.2, respectively)
PopulationE <- rnorm(12, 0, sqrt(1.2))
ContainerE <- rnorm(120, 0, sqrt(0.2))
# generation of response values on latent scale (!) based on fixed effects
# and random effects
ColourL <- with(DataMale, 0.8 * (-1) + 0.8 * (as.numeric(Treatment) - 1) + 0.5 *
    (as.numeric(Habitat) - 1) + PopulationE[Population] + ContainerE[Container])
# data generation (on data scale!) based on binomial distribution
DataMale$Colour <- rbinom(length(ColourL), 1, plogis(ColourL))
# looking at the data farme
str(DataMale)
```

```
## 'data.frame':    480 obs. of  6 variables:
##  $ Population: Factor w/ 12 levels "1","2","3","4",..: 1 1 1 1 1 1 1 1 1 1 ...
##  $ Container : Factor w/ 120 levels "1","2","3","4",..: 2 2 2 2 2 2 2 2 4 4 ...
##  $ Sex       : Factor w/ 2 levels "Female","Male": 2 2 2 2 2 2 2 2 2 2 ...
##  $ Habitat   : Factor w/ 2 levels "Dry","Wet": 1 1 1 1 2 2 2 2 1 1 ...
##  $ Treatment : Factor w/ 2 levels "Cont","Exp": 1 2 1 2 1 2 1 2 1 2 ...
##  $ Colour    : int  0 0 0 0 0 0 0 1 0 0 ...
```

Analysis

Preparation

We will use the four functions `glmmadmb`, `glmer` (`glmer.nb`), `glmmPQL` and `cpglmm` from the three `R` packages for analysis: `glmmADMB`, `lme4`, `MASS` and `cplm`, respectively. `MASS` should be already installed in the default `R` environment, but we will need to install the other two.

```
# installing glmmADMB
install.packages("R2admb")
install.packages("glmmADMB", repos = c("http://glmmadmb.r-forge.r-project.org/repos",
    getOption("repos")), type = "source")
# installing lme4
install.packages("lme4")
# installing cplm
install.packages("cplm")
```

We now load the four packages to be ready for analysis.

```
library(glmmADMB)
library(lme4)
library(MASS)
```

In the main text, we only report results from `glmmadmb`, but below, we will use at least two different functions to fit each of five different models below.

(1) Fecudity models: Quasi-Poisson GLMMs with log link

Below we use two founctions: `glmmabmb` and `glmmPQL`. They produce similar results both for regression coefficents (fixed effects) and variance components (random effects).

(1.1) `glmmadmb` fecundity models

```
# Fit null model without fixed effects (but including all random effects)
fecmodADMBr <- glmmadmb(Egg ~ 1 + (1 | Population) + (1 | Container), family = "nbinom1",
    data = DataFemale)
# Fit alternative model including fixed and all random effects
fecmodADMBf <- glmmadmb(Egg ~ Treatment + Habitat + (1 | Population) + (1 |
    Container), family = "nbinom1", data = DataFemale)
# View model fits for both models
summary(fecmodADMBr)
```

```
##
## Call:
## glmmadmb(formula = Egg ~ 1 + (1 | Population) + (1 | Container),
##     data = DataFemale, family = "nbinom1")
##
## AIC: 2498.8
##
## Coefficients:
##             Estimate Std. Error z value Pr(>|z|)
## (Intercept)    1.630      0.128    12.7   <2e-16 ***
## ---
## Signif. codes:  0 '***' 0.001 '**' 0.01 '*' 0.05 '.' 0.1 ' ' 1
##
## Number of observations: total=480, Population=12, Container=60
## Random effect variance(s):
## Group=Population
##             Variance StdDev
## (Intercept)   0.1775 0.4213
## Group=Container
##             Variance StdDev
## (Intercept)  0.04167 0.2041
##
## Negative binomial dispersion parameter: 2.2456 (std. err.: 0.16307)
##
## Log-likelihood: -1245.39
```

```
summary(fecmodADMBf)
```

```
##
## Call:
## glmmadmb(formula = Egg ~ Treatment + Habitat + (1 | Population) +
##     (1 | Container), data = DataFemale, family = "nbinom1")
##
## AIC: 2412.3
```

```
##
## Coefficients:
##              Estimate Std. Error z value Pr(>|z|)
## (Intercept)    1.2606     0.1384    9.11   <2e-16 ***
## TreatmentExp   0.4912     0.0511    9.61   <2e-16 ***
## HabitatWet     0.1518     0.0496    3.06   0.0022 **
## ---
## Signif. codes:  0 '***' 0.001 '**' 0.01 '*' 0.05 '.' 0.1 ' ' 1
##
## Number of observations: total=480, Population=12, Container=60
## Random effect variance(s):
## Group=Population
##             Variance StdDev
## (Intercept)   0.1873 0.4328
## Group=Container
##             Variance StdDev
## (Intercept)  0.05859 0.2421
##
## Negative binomial dispersion parameter: 1.7228 (std. err.: 0.12423)
##
## Log-likelihood: -1200.17
```

```
# Calculation of the variance in fitted values
VarF <- var(as.vector(model.matrix(fecmodADMBf) %*% fixef(fecmodADMBf)))

# getting the observation-level variance Null model
omegaN <- fecmodADMBr$alpha  # overdispersion omega is alpha in glmmadmb
lambda <- as.numeric(exp(fixef(fecmodADMBr) + 0.5 * (as.numeric(VarCorr(fecmodADMBr)[1]) +
    as.numeric(VarCorr(fecmodADMBr)[2]))))
# lambda2 <- mean(DataFemale$Egg) # for lambda we use the mean of all
# observations
VarOdN <- omegaN/lambda  # the delta method
VarOlN <- log(1 + omegaN/lambda)  # log-normal approximation
VarOtN <- trigamma(lambda/omegaN)  # trigamma function
# comparing the three
c(VarOdN = VarOdN, VarOlN = VarOlN, VarOlN = VarOtN)
```

```
##    VarOdN    VarOlN    VarOlN
## 0.3941326 0.3322724 0.4817193
```

```
# Full model
omegaF <- fecmodADMBf$alpha  # overdispersion omega is alpha in glmmadmb
VarOdF <- omegaF/lambda  # the delta method
VarOlF <- log(1 + omegaF/lambda)  # log-normal approximation
VarOtF <- trigamma(lambda/omegaF)  # trigamma function
# comparing the three
c(VarOdF = VarOdF, VarOlF = VarOlF, VarOlF = VarOtF)
```

```
##    VarOdF    VarOlF    VarOlF
## 0.3023743 0.2641890 0.3526177
```

```
# R2[GLMM(m)] - marginal R2[GLMM]
R2glmmM <- VarF/(VarF + sum(as.numeric(VarCorr(fecmodADMBf))) + VarOtF)
# R2[GLMM(c)] - conditional R2[GLMM] for full model
R2glmmC <- (VarF + sum(as.numeric(VarCorr(fecmodADMBf))))/(VarF + sum(as.numeric(VarCorr(fecmodADMBf)))
    VarOtF)
```

```r
# Raw unadjusted ICC[Population]
ICCrawPop <- VarCorr(fecmodADMBr)$Population[1]/(sum(as.numeric(VarCorr(fecmodADMBr))) +
    VarOtN)
# adjusted ICC[Population]
ICCadjPop <- VarCorr(fecmodADMBf)$Population[1]/(sum(as.numeric(VarCorr(fecmodADMBf))) +
    VarOtF)
# Raw unadjusted ICC[Container]
ICCrawCont <- VarCorr(fecmodADMBr)$Container[1]/(sum(as.numeric(VarCorr(fecmodADMBr))) +
    VarOtN)
# adjusted ICC[Container]
ICCadjCont <- VarCorr(fecmodADMBf)$Container[1]/(sum(as.numeric(VarCorr(fecmodADMBf))) +
    VarOtF)
# comparing the results
c(R2glmmM = R2glmmM, R2glmmC = R2glmmC, ICCrawPop = ICCrawPop, ICCadjPop = ICCadjPop,
    ICCrawCont = ICCrawCont, ICCadjCont = ICCadjCont)
```

```
##    R2glmmM    R2glmmC  ICCrawPop  ICCadjPop ICCrawCont ICCadjCont
## 0.09960058 0.46954680 0.25327172 0.31297893 0.05944866 0.09789008
```

(1.2) glmmPQL fecundity models

```r
# Fit null model without fixed effects (but including all random effects)
fecmodPQLr <- glmmPQL(Egg ~ 1, random = list(~1 | Population, ~1 | Container),
    family = "quasipoisson", data = DataFemale)
# Fit alternative model including fixed and all random effects
fecmodPQLf <- glmmPQL(Egg ~ Treatment + Habitat, random = list(~1 | Population,
    ~1 | Container), family = "quasipoisson", data = DataFemale)
# View model fits for both models
summary(fecmodPQLr)
```

```
## Linear mixed-effects model fit by maximum likelihood
##  Data: DataFemale
##   AIC BIC logLik
##    NA  NA     NA
##
## Random effects:
##  Formula: ~1 | Population
##         (Intercept)
## StdDev:   0.4404231
##
##  Formula: ~1 | Container %in% Population
##         (Intercept) Residual
## StdDev:   0.2394726 1.493101
##
## Variance function:
##  Structure: fixed weights
##  Formula: ~invwt
## Fixed effects: Egg ~ 1
##                Value Std.Error  DF  t-value p-value
## (Intercept) 1.627181  0.134709 420 12.07923       0
##
## Standardized Within-Group Residuals:
##        Min         Q1        Med         Q3        Max
```

```
## -2.0387389 -0.6771608 -0.2089840  0.5151110  5.3924954
##
## Number of Observations: 480
## Number of Groups:
##               Population Container %in% Population
##                       12                        60
```

```r
summary(fecmodPQLf)
```

```
## Linear mixed-effects model fit by maximum likelihood
##  Data: DataFemale
##   AIC BIC logLik
##    NA  NA     NA
##
## Random effects:
##  Formula: ~1 | Population
##         (Intercept)
## StdDev:   0.4397407
##
##  Formula: ~1 | Container %in% Population
##         (Intercept) Residual
## StdDev:   0.2555418 1.298306
##
## Variance function:
##  Structure: fixed weights
##  Formula: ~invwt
## Fixed effects: Egg ~ Treatment + Habitat
##                 Value  Std.Error  DF  t-value p-value
## (Intercept)  1.2500012 0.14078620 418  8.87872  0.0000
## TreatmentExp 0.5199280 0.05128843 418 10.13734  0.0000
## HabitatWet   0.1489874 0.04974055 418  2.99529  0.0029
##  Correlation:
##              (Intr) TrtmnE
## TreatmentExp -0.228
## HabitatWet   -0.190  0.000
##
## Standardized Within-Group Residuals:
##        Min         Q1        Med         Q3        Max
## -2.1075181 -0.6738357 -0.1211314  0.5105566  4.4290658
##
## Number of Observations: 480
## Number of Groups:
##               Population Container %in% Population
##                       12                        60
```

```r
# Calculation of the variance in fitted values
VarF <- var(as.vector(model.matrix(~Treatment + Habitat, data = DataFemale) %*%
    fixef(fecmodPQLf)))

# getting the observation-level variance Null model
omegaN <- as.numeric(VarCorr(fecmodPQLr)[5, 1])  # overdispersion omega is residual variance in glmmPQL
lambda <- as.numeric(exp(fixef(fecmodPQLr) + 0.5 * (as.numeric(VarCorr(fecmodPQLr)[2,
    1]) + as.numeric(VarCorr(fecmodPQLr)[4, 1]))))
# lambda2 <- mean(DataFemale$Egg)
VarOdN <- omegaN/lambda
```

```
VarOlN <- log(1 + omegaN/lambda)
VarOtN <- trigamma(lambda/omegaN)
# comparing the three
c(VarOdN = VarOdN, VarOlN = VarOlN, VarOtN = VarOtN)
```

```
##    VarOdN    VarOlN    VarOtN
## 0.3863041 0.3266413 0.4702665
```

```
# Full model
omegaF <- as.numeric(VarCorr(fecmodPQLf)[5, 1])  # overdispersion omega is residual variance in glmmPQL
VarOdF <- omegaF/lambda
VarOlF <- log(1 + omegaF/lambda)
VarOtF <- trigamma(lambda/omegaF)
# comparing the three
c(VarOdF = VarOdF, VarOlF = VarOlF, VarOtF = VarOtF)
```

```
##    VarOdF    VarOlF    VarOtF
## 0.2920823 0.2562551 0.3388243
```

```
# R2[GLMM(m)] - marginal R2[GLMM]
R2glmmM <- VarF/(VarF + sum(as.numeric(VarCorr(fecmodPQLf)[c(2, 4), 1])) + VarOtF)
# R2[GLMM(c)] - conditional R2[GLMM] for full model
R2glmmC <- (VarF + sum(as.numeric(VarCorr(fecmodPQLf)[c(2, 4), 1])))/(VarF +
    sum(as.numeric(VarCorr(fecmodPQLf)[c(2, 4), 1])) + VarOtF)
# Raw unadjusted ICC[Population]
ICCrawPop <- as.numeric(VarCorr(fecmodPQLr)[2, 1])/(sum(as.numeric(VarCorr(fecmodPQLr)[c(2,
    4), 1])) + VarOtN)
# adjusted ICC[Population]
ICCadjPop <- as.numeric(VarCorr(fecmodPQLf)[2, 1])/(sum(as.numeric(VarCorr(fecmodPQLf)[c(2,
    4), 1])) + VarOtF)
# Raw unadjusted ICC[Container]
ICCrawCont <- as.numeric(VarCorr(fecmodPQLr)[4, 1])/(sum(as.numeric(VarCorr(fecmodPQLr)[c(2,
    4), 1])) + VarOtN)
# adjusted ICC[Container]
ICCadjCont <- as.numeric(VarCorr(fecmodPQLf)[4, 1])/(sum(as.numeric(VarCorr(fecmodPQLf)[c(2,
    4), 1])) + VarOtF)
# comparing the results
c(R2glmmM = R2glmmM, R2glmmC = R2glmmC, ICCrawPop = ICCrawPop, ICCadjPop = ICCadjPop,
    ICCrawCont = ICCrawCont, ICCadjCont = ICCadjCont)
```

```
##    R2glmmM    R2glmmC  ICCrawPop  ICCadjPop ICCrawCont ICCadjCont
## 0.10925065 0.49488094 0.26881411 0.32363617 0.07947372 0.10929179
```

(2) Parasite models: Negative binomial GLMMs with log link

Below we use two founctions: `glmmabmb` and `glmer.nb`. They produce similar results both for regression coefficents (fixed effects) and variance components (random effects).

(2.1) `glmmadmb` parasite models

```
# Fit null model without fixed effects (but including all random effects)
parmodADMBr <- glmmadmb(Parasite ~ 1 + (1 | Population) + (1 | Container), family = "nbinom2",
    data = DataAll)
```

```r
# Fit alternative model including fixed and all random effects
parmodADMBf <- glmmadmb(Parasite ~ Sex + Treatment + Habitat + (1 | Population) +
    (1 | Container), family = "nbinom2", data = DataAll)
# View model fits for both models
summary(parmodADMBr)
```

```
##
## Call:
## glmmadmb(formula = Parasite ~ 1 + (1 | Population) + (1 | Container),
##     data = DataAll, family = "nbinom2")
##
## AIC: 4342.6
##
## Coefficients:
##             Estimate Std. Error z value Pr(>|z|)
## (Intercept)    0.766      0.222    3.45  0.00057 ***
## ---
## Signif. codes:  0 '***' 0.001 '**' 0.01 '*' 0.05 '.' 0.1 ' ' 1
##
## Number of observations: total=960, Population=12, Container=120
## Random effect variance(s):
## Group=Population
##             Variance StdDev
## (Intercept)   0.3745  0.612
## Group=Container
##             Variance StdDev
## (Intercept)    1.976  1.406
##
## Negative binomial dispersion parameter: 1.9848 (std. err.: 0.1725)
##
## Log-likelihood: -2167.31
```

```r
summary(parmodADMBf)
```

```
##
## Call:
## glmmadmb(formula = Parasite ~ Sex + Treatment + Habitat + (1 |
##     Population) + (1 | Container), data = DataAll, family = "nbinom2")
##
## AIC: 3920.5
##
## Coefficients:
##              Estimate Std. Error z value Pr(>|z|)
## (Intercept)    1.7524     0.2402    7.29    3e-13 ***
## SexMale       -2.1977     0.1599  -13.75   <2e-16 ***
## TreatmentExp  -0.7683     0.0518  -14.82   <2e-16 ***
## HabitatWet     0.7002     0.0516   13.57   <2e-16 ***
## ---
## Signif. codes:  0 '***' 0.001 '**' 0.01 '*' 0.05 '.' 0.1 ' ' 1
##
## Number of observations: total=960, Population=12, Container=120
## Random effect variance(s):
## Group=Population
##             Variance StdDev
```

```
## (Intercept)   0.5409 0.7354
## Group=Container
##              Variance StdDev
## (Intercept)   0.6128 0.7828
##
## Negative binomial dispersion parameter: 4.8684 (std. err.: 0.56935)
##
## Log-likelihood: -1953.25
```

```r
# Calculation of the variance in fitted values
VarF <- var(as.vector(model.matrix(parmodADMBf) %*% fixef(parmodADMBf)))

# getting the observation-level variance Null model
thetaN <- parmodADMBr$alpha  # note that theta is called alpha in glmmadmb
lambda <- as.numeric(exp(fixef(parmodADMBr) + 0.5 * (as.numeric(VarCorr(parmodADMBr)[1]) +
    as.numeric(VarCorr(parmodADMBr)[2]))))
# lambda2 <- mean(DataAll$Parasite)
VarOdN <- 1/lambda + 1/thetaN  # the delta method
VarOlN <- log(1 + (1/lambda) + (1/thetaN))  # log-normal approximation
VarOtN <- trigamma((1/lambda + 1/thetaN)^(-1))  # trigamma function
# comparing the three
c(VarOdN = VarOdN, VarOlN = VarOlN, VarOtN = VarOtN)
```

```
##    VarOdN    VarOlN    VarOtN
## 0.6473622 0.4991753 0.8990895
```

```r
# Full model
thetaF <- parmodADMBf$alpha  # note that theta is called alpha in glmmadmb
VarOdF <- 1/lambda + 1/thetaF  # the delta method
VarOlF <- log(1 + (1/lambda) + (1/thetaF))  # log-normal approximation
VarOtF <- trigamma((1/lambda + 1/thetaF)^(-1))  # trigamma function
# comparing the three
c(VarOdF = VarOdF, VarOlF = VarOlF, VarOtF = VarOtF)
```

```
##    VarOdF    VarOlF    VarOtF
## 0.3489394 0.2993187 0.4167403
```

```r
# R2[GLMM(m)] - marginal R2[GLMM]
R2glmmM <- VarF/(VarF + sum(as.numeric(VarCorr(parmodADMBf))) + VarOtF)
# R2[GLMM(c)] - conditional R2[GLMM] for full model
R2glmmC <- (VarF + sum(as.numeric(VarCorr(parmodADMBf))))/(VarF + sum(as.numeric(VarCorr(parmodADMBf)))
    VarOtF)
# Raw unadjusted ICC[Population]
ICCrawPop <- VarCorr(parmodADMBr)$Population[1]/(sum(as.numeric(VarCorr(parmodADMBr))) +
    VarOtN)
# adjusted ICC[Population]
ICCadjPop <- VarCorr(parmodADMBf)$Population[1]/(sum(as.numeric(VarCorr(parmodADMBf))) +
    VarOtF)
# Raw unadjusted ICC[Container]
ICCrawCont <- VarCorr(parmodADMBr)$Container[1]/(sum(as.numeric(VarCorr(parmodADMBr))) +
    VarOtN)
# adjusted ICC[Container]
ICCadjCont <- VarCorr(parmodADMBf)$Container[1]/(sum(as.numeric(VarCorr(parmodADMBf))) +
    VarOtF)
# comparing the results
c(R2glmmM = R2glmmM, R2glmmC = R2glmmC, ICCrawPop = ICCrawPop, ICCadjPop = ICCadjPop,
```

```r
    ICCrawCont = ICCrawCont, ICCadjCont = ICCadjCont)
```

```
##    R2glmmM    R2glmmC  ICCrawPop  ICCadjPop ICCrawCont ICCadjCont
##  0.4850407  0.8633433  0.1152660  0.3444048  0.6080433  0.3902215
```

(2.2) glmer.nb parasite models

```r
# Fit null model without fixed effects (but including all random effects)
parmodGLMERr <- glmer.nb(Parasite ~ 1 + (1 | Population) + (1 | Container),
    data = DataAll)
# Fit alternative model including fixed and all random effects
parmodGLMERf <- glmer.nb(Parasite ~ Sex + Treatment + Habitat + (1 | Population) +
    (1 | Container), data = DataAll)
# View model fits for both models
summary(parmodGLMERr)
```

```
## Generalized linear mixed model fit by maximum likelihood (Laplace
##   Approximation) [glmerMod]
##  Family: Negative Binomial(1.9844)  ( log )
## Formula: Parasite ~ 1 + (1 | Population) + (1 | Container)
##    Data: DataAll
##
##      AIC      BIC   logLik deviance df.resid
##   4342.5   4361.9  -2167.2   4334.5      956
##
## Scaled residuals:
##     Min      1Q  Median      3Q     Max
## -1.3006 -0.6760 -0.3297  0.4313  4.0466
##
## Random effects:
##  Groups     Name        Variance Std.Dev.
##  Container  (Intercept) 1.9734   1.4048
##  Population (Intercept) 0.3718   0.6098
## Number of obs: 960, groups:  Container, 120; Population, 12
##
## Fixed effects:
##             Estimate Std. Error z value Pr(>|z|)
## (Intercept)   0.7368     0.2217   3.323  0.00089 ***
## ---
## Signif. codes:  0 '***' 0.001 '**' 0.01 '*' 0.05 '.' 0.1 ' ' 1
```

```r
summary(parmodGLMERf)
```

```
## Generalized linear mixed model fit by maximum likelihood (Laplace
##   Approximation) [glmerMod]
##  Family: Negative Binomial(4.8664)  ( log )
## Formula: Parasite ~ Sex + Treatment + Habitat + (1 | Population) + (1 |
##     Container)
##    Data: DataAll
##
##      AIC      BIC   logLik deviance df.resid
##   3920.5   3954.5  -1953.2   3906.5      953
##
```

```
## Scaled residuals:
##     Min      1Q  Median      3Q     Max
## -1.8116 -0.6497 -0.2998  0.5259  3.3393
##
## Random effects:
##  Groups     Name        Variance Std.Dev.
##  Container  (Intercept) 0.6124   0.7826
##  Population (Intercept) 0.5393   0.7344
## Number of obs: 960, groups:  Container, 120; Population, 12
##
## Fixed effects:
##              Estimate Std. Error z value Pr(>|z|)
## (Intercept)   1.74034    0.23993   7.254 4.06e-13 ***
## SexMale      -2.19616    0.15978 -13.745  < 2e-16 ***
## TreatmentExp -0.76439    0.05156 -14.825  < 2e-16 ***
## HabitatWet    0.69646    0.05135  13.564  < 2e-16 ***
## ---
## Signif. codes:  0 '***' 0.001 '**' 0.01 '*' 0.05 '.' 0.1 ' ' 1
##
## Correlation of Fixed Effects:
##             (Intr) SexMal TrtmnE
## SexMale     -0.292
## TreatmntExp -0.088  0.023
## HabitatWet  -0.123 -0.021 -0.007
```

```
# Calculation of the variance in fitted values
VarF <- var(as.vector(model.matrix(parmodGLMERf) %*% fixef(parmodGLMERf)))

# getting the observation-level variance Null model
thetaN <- getME(parmodGLMERr, "glmer.nb.theta")
lambda <- as.numeric(exp(fixef(parmodGLMERr) + 0.5 * (as.numeric(VarCorr(parmodGLMERr)$Population) +
    as.numeric(VarCorr(parmodGLMERr)$Container))))
# lambda2 <- mean(DataAll$Parasite)
VarOdN <- 1/lambda + 1/thetaN  # the delta method
VarOlN <- log(1 + (1/lambda) + (1/thetaN))  # log-normal approximation
VarOtN <- trigamma((1/lambda + 1/thetaN)^(-1))  # trigamma function
# comparing the three
c(VarOdN = VarOdN, VarOlN = VarOlN, VarOtN = VarOtN)
```

```
##    VarOdN    VarOlN    VarOtN
## 0.6520918 0.5020423 0.9077864
```

```
# Full model
thetaF <- getME(parmodGLMERf, "glmer.nb.theta")
VarOdF <- 1/lambda + 1/thetaF  # the delta method
VarOlF <- log(1 + (1/lambda) + (1/thetaF))  # log-normal approximation
VarOtF <- trigamma((1/lambda + 1/thetaF)^(-1))  # trigamma function
# comparing the three
c(VarOdF = VarOdF, VarOlF = VarOlF, VarOtF = VarOtF)
```

```
##    VarOdF    VarOlF    VarOtF
## 0.3536557 0.3028088 0.4233937
```

```
# R2[GLMM(m)] - marginal R2[GLMM]
R2glmmM <- VarF/(VarF + sum(as.numeric(VarCorr(parmodGLMERf))) + VarOtF)
# R2[GLMM(c)] - conditional R2[GLMM] for full model
```

```r
R2glmmC <- (VarF + sum(as.numeric(VarCorr(parmodGLMERf))))/(VarF + sum(as.numeric(VarCorr(parmodGLMERf)
    VarOtF)
# Raw unadjusted ICC[Population]
ICCrawPop <- as.numeric(VarCorr(parmodGLMERr)$Population)/(sum(as.numeric(VarCorr(parmodGLMERr))) +
    VarOtN)
# adjusted ICC[Population]
ICCadjPop <- as.numeric(VarCorr(parmodGLMERf)$Population)/(sum(as.numeric(VarCorr(parmodGLMERf))) +
    VarOtF)
# Raw unadjusted ICC[Container]
ICCrawCont <- as.numeric(VarCorr(parmodGLMERr)$Container)/(sum(as.numeric(VarCorr(parmodGLMERr))) +
    VarOtN)
# adjusted ICC[Container]
ICCadjCont <- as.numeric(VarCorr(parmodGLMERf)$Container)/(sum(as.numeric(VarCorr(parmodGLMERf))) +
    VarOtF)
# comparing the results
c(R2glmmM = R2glmmM, R2glmmC = R2glmmC, ICCrawPop = ICCrawPop, ICCadjPop = ICCadjPop,
    ICCrawCont = ICCrawCont, ICCadjCont = ICCadjCont)
```

```
##    R2glmmM    R2glmmC  ICCrawPop  ICCadjPop ICCrawCont ICCadjCont
##  0.4835241  0.8611736  0.1143047  0.3423972  0.6066358  0.3888073
```

(3) Size models: Gamma GLMMs with log link

Below we use three founctions: `glmmabmb`, `glmmPQL` and `glmer`. They produce similar results both for regression coefficents but not for variance components (random effects). The `glmer` variance components were different form the others.

(3.1) `glmmadmb` size models

```r
# Fit null model without fixed effects (but including all random effects)
sizemodADMBr <- glmmadmb(BodyL ~ 1 + (1 | Population) + (1 | Container), family = "gamma",
    data = DataAll)
# Fit alternative model including fixed and all random effects
sizemodADMBf <- glmmadmb(BodyL ~ Sex + Treatment + Habitat + (1 | Population) +
    (1 | Container), family = "gamma", data = DataAll)
# View model fits for both models
summary(sizemodADMBr)
```

```
##
## Call:
## glmmadmb(formula = BodyL ~ 1 + (1 | Population) + (1 | Container),
##     data = DataAll, family = "gamma")
##
## AIC: 3379.9
##
## Coefficients:
##             Estimate Std. Error z value Pr(>|z|)
## (Intercept)   2.6522     0.0186     143   <2e-16 ***
## ---
## Signif. codes:  0 '***' 0.001 '**' 0.01 '*' 0.05 '.' 0.1 ' ' 1
##
## Number of observations: total=960, Population=12, Container=120
```

```
## Random effect variance(s):
## Group=Population
##             Variance  StdDev
## (Intercept) 0.002666 0.05163
## Group=Container
##             Variance StdDev
## (Intercept)  0.01397 0.1182
##
## Gamma shape parameter: 147.88 (std. err.: 7.2064)
##
## Log-likelihood: -1685.93
```

```r
summary(sizemodADMBf)
```

```
##
## Call:
## glmmadmb(formula = BodyL ~ Sex + Treatment + Habitat + (1 | Population) +
##     (1 | Container), data = DataAll, family = "gamma")
##
## AIC: 3139.5
##
## Coefficients:
##              Estimate Std. Error z value Pr(>|z|)
## (Intercept)   2.73720    0.01944  140.77  < 2e-16 ***
## SexMale      -0.21265    0.00869  -24.46  < 2e-16 ***
## TreatmentExp  0.03340    0.00517    6.46  1.1e-10 ***
## HabitatWet    0.00901    0.00517    1.74    0.082 .
## ---
## Signif. codes:  0 '***' 0.001 '**' 0.01 '*' 0.05 '.' 0.1 ' ' 1
##
## Number of observations: total=960, Population=12, Container=120
## Random effect variance(s):
## Group=Population
##             Variance  StdDev
## (Intercept) 0.003925 0.06265
## Group=Container
##             Variance  StdDev
## (Intercept) 0.001464 0.03826
##
## Gamma shape parameter: 155.72 (std. err.: 7.5896)
##
## Log-likelihood: -1562.77
```

```r
# Calculation of the variance in fitted values
VarF <- var(as.vector(model.matrix(sizemodADMBf) %*% fixef(sizemodADMBf)))

# getting the observation-level variance Null model
nuN <- sizemodADMBr$alpha  # overdispersion omega is alpha in glmmadmb
VarOdN <- 1/nuN  # the delta method
VarOlN <- log(1 + 1/nuN)  # log-normal approximation
VarOtN <- trigamma(nuN)  # trigamma function
# comparing the three
c(VarOdN = VarOdN, VarOlN = VarOlN, VarOtN = VarOtN)
```

```
##      VarOdN      VarOlN      VarOtN
```

```
## 0.006762240 0.006739478 0.006785155
```

```r
# Full model
nuF <- sizemodADMBf$alpha  # overdispersion omega is alpha in glmmadmb
VarOdF <- 1/nuF  # the delta method
VarOlF <- log(1 + 1/nuF)  # log-normal approximation
VarOtF <- trigamma(nuF)  # trigamma function
# comparing the three
c(VarOdF = VarOdF, VarOlF = VarOlF, VarOtF = VarOtF)
```

```
##      VarOdF      VarOlF      VarOtF
## 0.006421783 0.006401251 0.006442446
```

```r
# R2[GLMM(m)] - marginal R2[GLMM]
R2glmmM <- VarF/(VarF + sum(as.numeric(VarCorr(sizemodADMBf))) + VarOtF)
# R2[GLMM(c)] - conditional R2[GLMM] for full model
R2glmmC <- (VarF + sum(as.numeric(VarCorr(sizemodADMBf))))/(VarF + sum(as.numeric(VarCorr(sizemodADMBf)
    VarOtF)
# Raw unadjusted ICC[Population]
ICCrawPop <- VarCorr(sizemodADMBr)$Population[1]/(sum(as.numeric(VarCorr(sizemodADMBr))) +
    VarOtN)
# adjusted ICC[Population]
ICCadjPop <- VarCorr(sizemodADMBf)$Population[1]/(sum(as.numeric(VarCorr(sizemodADMBf))) +
    VarOtF)
# Raw unadjusted ICC[Container]
ICCrawCont <- VarCorr(sizemodADMBr)$Container[1]/(sum(as.numeric(VarCorr(sizemodADMBr))) +
    VarOtN)
# adjusted ICC[Container]
ICCadjCont <- VarCorr(sizemodADMBf)$Container[1]/(sum(as.numeric(VarCorr(sizemodADMBf))) +
    VarOtF)
# comparing the results
c(R2glmmM = R2glmmM, R2glmmC = R2glmmC, ICCrawPop = ICCrawPop, ICCadjPop = ICCadjPop,
    ICCrawCont = ICCrawCont, ICCadjCont = ICCadjCont)
```

```
##   R2glmmM   R2glmmC  ICCrawPop  ICCadjPop ICCrawCont ICCadjCont
##  0.4954184  0.7252318  0.1137893  0.3317261  0.5965656  0.1237274
```

(3.2) glmmPQL size models

```r
# Fit null model without fixed effects (but including all random effects)
sizemodPQLr <- glmmPQL(BodyL ~ 1, random = list(~1 | Population, ~1 | Container),
    family = Gamma(link = log), data = DataAll)
# Fit alternative model including fixed and all random effects
sizemodPQLf <- glmmPQL(BodyL ~ Sex + Treatment + Habitat, random = list(~1 |
    Population, ~1 | Container), family = Gamma(link = log), data = DataAll)
# View model fits for both models
summary(sizemodPQLr)
```

```
## Linear mixed-effects model fit by maximum likelihood
##  Data: DataAll
##   AIC BIC logLik
##    NA  NA     NA
##
## Random effects:
##  Formula: ~1 | Population
```

```
##         (Intercept)
## StdDev:  0.05163009
##
##  Formula: ~1 | Container %in% Population
##         (Intercept)   Residual
## StdDev:   0.1182651 0.08168051
##
## Variance function:
##  Structure: fixed weights
##  Formula: ~invwt
## Fixed effects: BodyL ~ 1
##                Value  Std.Error  DF  t-value p-value
## (Intercept) 2.651846 0.01860119 840 142.5633       0
##
## Standardized Within-Group Residuals:
##         Min          Q1         Med          Q3         Max
## -3.03349978 -0.62464740  0.05334217  0.66652467  2.94836006
##
## Number of Observations: 960
## Number of Groups:
##                Population Container %in% Population
##                        12                       120
```

```
summary(sizemodPQLf)
```

```
## Linear mixed-effects model fit by maximum likelihood
##  Data: DataAll
##   AIC BIC logLik
##    NA  NA     NA
##
## Random effects:
##  Formula: ~1 | Population
##         (Intercept)
## StdDev:  0.06264751
##
##  Formula: ~1 | Container %in% Population
##         (Intercept)   Residual
## StdDev:  0.03841503 0.07954664
##
## Variance function:
##  Structure: fixed weights
##  Formula: ~invwt
## Fixed effects: BodyL ~ Sex + Treatment + Habitat
##                   Value   Std.Error  DF   t-value p-value
## (Intercept)   2.7369180 0.019483361 838 140.47463  0.0000
## SexMale      -0.2126482 0.008710447 107 -24.41300  0.0000
## TreatmentExp  0.0333985 0.005145444 838   6.49089  0.0000
## HabitatWet    0.0090129 0.005145444 838   1.75162  0.0802
##  Correlation:
##              (Intr) SexMal TrtmnE
## SexMale      -0.224
## TreatmentExp -0.132  0.000
## HabitatWet   -0.132  0.000  0.000
##
## Standardized Within-Group Residuals:
```

```
##        Min         Q1        Med         Q3        Max 
## -2.9532554 -0.5984901  0.0251208  0.6208741  2.7806472 
## 
## Number of Observations: 960
## Number of Groups: 
##               Population Container %in% Population 
##                       12                        120
```

```
# Calculation of the variance in fitted values
VarF <- var(as.vector(model.matrix(~Sex + Treatment + Habitat, data = DataAll) %*%
    fixef(sizemodPQLf)))

# getting the observation-level variance Null model
nuN <- 1/as.numeric(VarCorr(sizemodPQLr)[5, 1])  # note that glmmPQL report 1/nu not nu as resiudal var
VarOdN <- 1/nuN  # the delta method
VarOlN <- log(1 + 1/nuN)  # log-normal approximation
VarOtN <- trigamma(nuN)  # trigamma function
# comparing the three
c(VarOdN = VarOdN, VarOlN = VarOlN, VarOtN = VarOtN)
```

```
##      VarOdN      VarOlN      VarOtN 
## 0.006671705 0.006649548 0.006694010
```

```
# Full model
nuF <- 1/as.numeric(VarCorr(sizemodPQLf)[5, 1])  # note that glmmPQL report 1/nu not nu as resiudal var
VarOdF <- 1/nuF  # the delta method
VarOlF <- log(1 + 1/nuF)  # log-normal approximation
VarOtF <- trigamma(nuF)  # trigamma function
# comparing the three
c(VarOdF = VarOdF, VarOlF = VarOlF, VarOtF = VarOtF)
```

```
##      VarOdF      VarOlF      VarOtF 
## 0.006327667 0.006307731 0.006347729
```

```
# R2[GLMM(m)] - marginal R2[GLMM]
R2glmmM <- VarF/(VarF + sum(as.numeric(VarCorr(sizemodPQLf)[c(2, 4), 1])) +
    VarOtF)
# R2[GLMM(c)] - conditional R2[GLMM] for full model
R2glmmC <- (VarF + sum(as.numeric(VarCorr(sizemodPQLf)[c(2, 4), 1])))/(VarF +
    sum(as.numeric(VarCorr(sizemodPQLf)[c(2, 4), 1])) + VarOtF)
# Raw unadjusted ICC[Population]
ICCrawPop <- as.numeric(VarCorr(sizemodPQLr)[2, 1])/(sum(as.numeric(VarCorr(sizemodPQLr)[c(2,
    4), 1])) + VarOtN)
# adjusted ICC[Population]
ICCadjPop <- as.numeric(VarCorr(sizemodPQLf)[2, 1])/(sum(as.numeric(VarCorr(sizemodPQLf)[c(2,
    4), 1])) + VarOtF)
# Raw unadjusted ICC[Container]
ICCrawCont <- as.numeric(VarCorr(sizemodPQLr)[4, 1])/(sum(as.numeric(VarCorr(sizemodPQLr)[c(2,
    4), 1])) + VarOtN)
# adjusted ICC[Container]
ICCadjCont <- as.numeric(VarCorr(sizemodPQLf)[4, 1])/(sum(as.numeric(VarCorr(sizemodPQLf)[c(2,
    4), 1])) + VarOtF)
# comparing the results
c(R2glmmM = R2glmmM, R2glmmC = R2glmmC, ICCrawPop = ICCrawPop, ICCadjPop = ICCadjPop,
    ICCrawCont = ICCrawCont, ICCadjCont = ICCadjCont)
```

```
##    R2glmmM    R2glmmC  ICCrawPop  ICCadjPop ICCrawCont ICCadjCont
##  0.4971737  0.7283143  0.1141794  0.3340704  0.5990938  0.1256124
```

(3.3) glmer size models

```r
# Fit null model without fixed effects (but including all random effects)
sizemodeGLMERr <- glmer(BodyL ~ 1 + (1 | Population) + (1 | Container), family = Gamma(link = log),
    data = DataAll)
# Fit alternative model including fixed and all random effects
sizemodeGLMERf <- glmer(BodyL ~ Sex + Treatment + Habitat + (1 | Population) +
    (1 | Container), family = Gamma(link = log), data = DataAll)
# View model fits for both models
summary(sizemodeGLMERr)
```

```
## Generalized linear mixed model fit by maximum likelihood (Laplace
##   Approximation) [glmerMod]
##  Family: Gamma  ( log )
## Formula: BodyL ~ 1 + (1 | Population) + (1 | Container)
##    Data: DataAll
##
##      AIC      BIC   logLik deviance df.resid
##   3185.5   3205.0  -1588.7   3177.5      956
##
## Scaled residuals:
##      Min       1Q   Median       3Q      Max
## -2.83568 -0.55540  0.05456  0.60620  2.62809
##
## Random effects:
##  Groups     Name        Variance  Std.Dev.
##  Container  (Intercept) 0.0054135 0.07358
##  Population (Intercept) 0.0004434 0.02106
##  Residual               0.0083710 0.09149
## Number of obs: 960, groups:  Container, 120; Population, 12
##
## Fixed effects:
##             Estimate Std. Error t value Pr(>|z|)
## (Intercept)  2.64882    0.02596     102   <2e-16 ***
## ---
## Signif. codes:  0 '***' 0.001 '**' 0.01 '*' 0.05 '.' 0.1 ' ' 1
```

```r
summary(sizemodeGLMERf)
```

```
## Generalized linear mixed model fit by maximum likelihood (Laplace
##   Approximation) [glmerMod]
##  Family: Gamma  ( log )
## Formula:
## BodyL ~ Sex + Treatment + Habitat + (1 | Population) + (1 | Container)
##    Data: DataAll
##
##      AIC      BIC   logLik deviance df.resid
##   3042.2   3076.2  -1514.1   3028.2      953
##
## Scaled residuals:
##      Min       1Q   Median       3Q      Max
```

```
## -2.97652 -0.58856  0.02293  0.62893  2.62252
##
## Random effects:
##  Groups     Name        Variance  Std.Dev.
##  Container  (Intercept) 0.0012338 0.03513
##  Population (Intercept) 0.0006937 0.02634
##  Residual               0.0067507 0.08216
## Number of obs: 960, groups:  Container, 120; Population, 12
##
## Fixed effects:
##              Estimate Std. Error t value Pr(>|z|)
## (Intercept)   2.734713   0.026910  101.62  < 2e-16 ***
## SexMale      -0.212780   0.012255  -17.36  < 2e-16 ***
## TreatmentExp  0.033385   0.004999    6.68 2.41e-11 ***
## HabitatWet    0.009007   0.004999    1.80   0.0716 .
## ---
## Signif. codes:  0 '***' 0.001 '**' 0.01 '*' 0.05 '.' 0.1 ' ' 1
##
## Correlation of Fixed Effects:
##             (Intr) SexMal TrtmnE
## SexMale     -0.227
## TreatmntExp -0.092 -0.001
## HabitatWet  -0.092  0.000 -0.004
```

```
# Calculation of the variance in fitted values
VarF <- var(as.vector(model.matrix(sizemodeGLMERf) %*% fixef(sizemodeGLMERf)))

# getting the observation-level variance Null model
nuN <- 1/attr(VarCorr(sizemodeGLMERr), "sc")^2  # note that glmer report 1/nu not nu as resiudal varian
VarOdN <- 1/nuN  # the delta method
VarOlN <- log(1 + 1/nuN)  # log-normal approximation
VarOtN <- trigamma(nuN)  # trigamma function
# comparing the three
c(VarOdN = VarOdN, VarOlN = VarOlN, VarOtN = VarOtN)
```

```
##      VarOdN      VarOlN      VarOtN
## 0.008370998 0.008336156 0.008406133
```

```
# Full model
nuF <- 1/attr(VarCorr(sizemodeGLMERf), "sc")^2  # note that glmer report 1/nu not nu as resiudal varian
VarOdF <- 1/nuF  # the delta method
VarOlF <- log(1 + 1/nuF)  # log-normal approximation
VarOtF <- trigamma(nuF)  # trigamma function
# comparing the three
c(VarOdF = VarOdF, VarOlF = VarOlF, VarOtF = VarOtF)
```

```
##      VarOdF      VarOlF      VarOtF
## 0.006750704 0.006728020 0.006773541
```

```
# R2[GLMM(m)] - marginal R2[GLMM]
R2glmmM <- VarF/(VarF + sum(as.numeric(VarCorr(sizemodeGLMERf))) + VarOtF)
# R2[GLMM(c)] - conditional R2[GLMM] for full model
R2glmmC <- (VarF + sum(as.numeric(VarCorr(sizemodeGLMERf))))/(VarF + sum(as.numeric(VarCorr(sizemodeGLM
    VarOtF)
# Raw unadjusted ICC[Population]
ICCrawPop <- as.numeric(VarCorr(sizemodeGLMERr)$Population)/(sum(as.numeric(VarCorr(sizemodeGLMERr))) +
```

```
    VarOtN)
# adjusted ICC[Population]
ICCadjPop <- as.numeric(VarCorr(sizemodeGLMERf)$Population)/(sum(as.numeric(VarCorr(sizemodeGLMERf))) +
    VarOtF)
# Raw unadjusted ICC[Container]
ICCrawCont <- as.numeric(VarCorr(sizemodeGLMERr)$Container)/(sum(as.numeric(VarCorr(sizemodeGLMERr))) +
    VarOtN)
# adjusted ICC[Container]
ICCadjCont <- as.numeric(VarCorr(sizemodeGLMERf)$Container)/(sum(as.numeric(VarCorr(sizemodeGLMERf))) +
    VarOtF)
# comparing the results
c(R2glmmM = R2glmmM, R2glmmC = R2glmmC, ICCrawPop = ICCrawPop, ICCadjPop = ICCadjPop,
    ICCrawCont = ICCrawCont, ICCadjCont = ICCadjCont)
```

```
##    R2glmmM    R2glmmC  ICCrawPop  ICCadjPop ICCrawCont ICCadjCont
## 0.57202668 0.66683554 0.03109067 0.07972609 0.37954437 0.14180373
```

(4) Exploration models: Gamma GLMMs with log link

Below we use three founctions: `glmmabmb`, `glmmPQL` and `glmer`. As for the previous section, they produce similar results both for regression coefficents but not for variance components (random effects). The `glmer` variance components were different form the others.

(4.1) `glmmadmb` exploration models

```
# Fit null model without fixed effects (but including all random effects)
explmodADMBr <- glmmadmb(Exploration ~ 1 + (1 | Population) + (1 | Container),
    family = "gamma", data = DataAll)
# Fit alternative model including fixed and all random effects
explmodADMBf <- glmmadmb(Exploration ~ Sex + Treatment + Habitat + (1 | Population) +
    (1 | Container), family = "gamma", data = DataAll)
# View model fits for both models
summary(explmodADMBr)
```

```
##
## Call:
## glmmadmb(formula = Exploration ~ 1 + (1 | Population) + (1 |
##     Container), data = DataAll, family = "gamma")
##
## AIC: 11223.8
##
## Coefficients:
##             Estimate Std. Error z value Pr(>|z|)
## (Intercept)    4.752      0.101    47.3   <2e-16 ***
## ---
## Signif. codes:  0 '***' 0.001 '**' 0.01 '*' 0.05 '.' 0.1 ' ' 1
##
## Number of observations: total=960, Population=12, Container=120
## Random effect variance(s):
## Group=Population
##             Variance StdDev
## (Intercept)  0.07137 0.2672
```

```
## Group=Container
##             Variance StdDev
## (Intercept)   0.3639 0.6032
##
## Gamma shape parameter: 0.99229 (std. err.: 0.042061)
##
## Log-likelihood: -5607.89
```

```r
summary(explmodADMBf)
```

```
##
## Call:
## glmmadmb(formula = Exploration ~ Sex + Treatment + Habitat +
##     (1 | Population) + (1 | Container), data = DataAll, family = "gamma")
##
## AIC: 9004.3
##
## Coefficients:
##              Estimate Std. Error z value Pr(>|z|)
## (Intercept)    4.0556     0.1088    37.3   <2e-16 ***
## SexMale       -1.1051     0.0768   -14.4   <2e-16 ***
## TreatmentExp   2.0076     0.0217    92.3   <2e-16 ***
## HabitatWet    -0.5604     0.0217   -25.8   <2e-16 ***
## ---
## Signif. codes:  0 '***' 0.001 '**' 0.01 '*' 0.05 '.' 0.1 ' ' 1
##
## Number of observations: total=960, Population=12, Container=120
## Random effect variance(s):
## Group=Population
##             Variance StdDev
## (Intercept)   0.1037 0.3221
## Group=Container
##             Variance StdDev
## (Intercept)    0.163 0.4038
##
## Gamma shape parameter: 8.9434 (std. err.: 0.42749)
##
## Log-likelihood: -4495.13
```

```r
# Calculation of the variance in fitted values
VarF <- var(as.vector(model.matrix(explmodADMBf) %*% fixef(explmodADMBf)))

# getting the observation-level variance Null model
nuN <- explmodADMBr$alpha  #note nu theta is called alpha in glmmadmb
VarOdN <- 1/nuN  # the delta method
VarOlN <- log(1 + 1/nuN)  # log-normal approximation
VarOtN <- trigamma(nuN)  # trigamma function
# comparing the three
c(VarOdN = VarOdN, VarOlN = VarOlN, VarOtN = VarOtN)
```

```
##    VarOdN    VarOlN    VarOtN
## 1.0077699 0.6970246 1.6636647
```

```r
# Full model
nuF <- explmodADMBf$alpha  #note nu theta is called alpha in glmmadmb
VarOdF <- 1/nuF  # the delta method
```

```r
Var0lF <- log(1 + 1/nuF)  # log-normal approximation
Var0tF <- trigamma(nuF)  # trigamma function
# comparing the three
c(Var0dF = Var0dF, Var0lF = Var0lF, Var0tF = Var0tF)
```

```
##    Var0dF    Var0lF    Var0tF
## 0.1118143 0.1059932 0.1182979
```

```r
# R2[GLMM(m)] - marginal R2[GLMM]
R2glmmM <- VarF/(VarF + sum(as.numeric(VarCorr(explmodADMBf))) + Var0tF)
# R2[GLMM(c)] - conditional R2[GLMM] for full model
R2glmmC <- (VarF + sum(as.numeric(VarCorr(explmodADMBf))))/(VarF + sum(as.numeric(VarCorr(explmodADMBf))
    Var0tF)
# Raw unadjusted ICC[Population]
ICCrawPop <- VarCorr(explmodADMBr)$Population[1]/(sum(as.numeric(VarCorr(explmodADMBr))) +
    Var0tN)
# adjusted ICC[Population]
ICCadjPop <- VarCorr(explmodADMBf)$Population[1]/(sum(as.numeric(VarCorr(explmodADMBf))) +
    Var0tF)
# Raw unadjusted ICC[Container]
ICCrawCont <- VarCorr(explmodADMBr)$Container[1]/(sum(as.numeric(VarCorr(explmodADMBr))) +
    Var0tN)
# adjusted ICC[Container]
ICCadjCont <- VarCorr(explmodADMBf)$Container[1]/(sum(as.numeric(VarCorr(explmodADMBf))) +
    Var0tF)
# comparing the results
c(R2glmmM = R2glmmM, R2glmmC = R2glmmC, ICCrawPop = ICCrawPop, ICCadjPop = ICCadjPop,
    ICCrawCont = ICCrawCont, ICCadjCont = ICCadjCont)
```

```
##    R2glmmM    R2glmmC  ICCrawPop  ICCadjPop ICCrawCont ICCadjCont
## 0.78342883 0.93346476 0.03400497 0.26938804 0.17336939 0.42339084
```

(4.2) glmPQL exploration models

```r
# Fit null model without fixed effects (but including all random effects)
explmodPQLr <- glmmPQL(Exploration ~ 1, random = list(~1 | Population, ~1 |
    Container), family = Gamma(link = log), data = DataAll)
# Fit alternative model including fixed and all random effects
explmodPQLf <- glmmPQL(Exploration ~ Sex + Treatment + Habitat, random = list(~1 |
    Population, ~1 | Container), family = Gamma(link = log), data = DataAll)
# View model fits for both models
summary(explmodPQLr)
```

```
## Linear mixed-effects model fit by maximum likelihood
##  Data: DataAll
##   AIC BIC logLik
##    NA  NA     NA
##
## Random effects:
##  Formula: ~1 | Population
##         (Intercept)
## StdDev:   0.2722396
##
##  Formula: ~1 | Container %in% Population
```

```
##         (Intercept)  Residual
## StdDev:   0.6292432 0.9028776
##
## Variance function:
##  Structure: fixed weights
##  Formula: ~invwt
## Fixed effects: Exploration ~ 1
##                Value Std.Error  DF  t-value p-value
## (Intercept) 4.697597 0.1016645 840 46.20684       0
##
## Standardized Within-Group Residuals:
##        Min         Q1        Med         Q3        Max
## -1.0973802 -0.8508788 -0.4618470  0.7489602  2.2125998
##
## Number of Observations: 960
## Number of Groups:
##                Population Container %in% Population
##                        12                       120
```

```r
summary(explmodPQLf)
```

```
## Linear mixed-effects model fit by maximum likelihood
##  Data: DataAll
##   AIC BIC logLik
##    NA  NA     NA
##
## Random effects:
##  Formula: ~1 | Population
##         (Intercept)
## StdDev:   0.3221272
##
##  Formula: ~1 | Container %in% Population
##         (Intercept)  Residual
## StdDev:   0.4061742 0.3123817
##
## Variance function:
##  Structure: fixed weights
##  Formula: ~invwt
## Fixed effects: Exploration ~ Sex + Treatment + Habitat
##                  Value  Std.Error  DF   t-value p-value
## (Intercept)   4.048990 0.10887040 838  37.19092       0
## SexMale      -1.105110 0.07701008 107 -14.35021       0
## TreatmentExp  2.007820 0.02020630 838  99.36606       0
## HabitatWet   -0.560711 0.02020630 838 -27.74930       0
##  Correlation:
##              (Intr) SexMal TrtmnE
## SexMale      -0.354
## TreatmentExp -0.093  0.000
## HabitatWet   -0.093  0.000  0.000
##
## Standardized Within-Group Residuals:
##         Min          Q1         Med          Q3         Max
## -3.03017476 -0.48055376 -0.03782469  0.49816646  5.78965007
##
## Number of Observations: 960
```

```
## Number of Groups:
##                 Population Container %in% Population
##                         12                        120
```

```r
# Calculation of the variance in fitted values
VarF <- var(as.vector(model.matrix(~Sex + Treatment + Habitat, data = DataAll) %*%
    fixef(explmodPQLf)))

# getting the observation-level variance Null model
nuN <- 1/as.numeric(VarCorr(explmodPQLr)[5, 1])  # note that glmmPQL report 1/nu not nu as resiudal var
VarOdN <- 1/nuN  # the delta method
VarOlN <- log(1 + 1/nuN)  # log-normal approximation
VarOtN <- trigamma(nuN)  # trigamma function
# comparing the three
c(VarOdN = VarOdN, VarOlN = VarOlN, VarOtN = VarOtN)
```

```
##   VarOdN   VarOlN   VarOtN
## 0.815188 0.596189 1.229023
```

```r
# Full model
nuF <- 1/as.numeric(VarCorr(explmodPQLf)[5, 1])  # note that glmmPQL report 1/nu not nu as resiudal var
VarOdF <- 1/nuF  # the delta method
VarOlF <- log(1 + 1/nuF)  # log-normal approximation
VarOtF <- trigamma(nuF)  # trigamma function
# comparing the three
c(VarOdF = VarOdF, VarOlF = VarOlF, VarOtF = VarOtF)
```

```
##     VarOdF     VarOlF     VarOtF
## 0.09758235 0.09310990 0.10249808
```

```r
# R2[GLMM(m)] - marginal R2[GLMM]
R2glmmM <- VarF/(VarF + sum(as.numeric(VarCorr(explmodPQLf)[c(2, 4), 1])) +
    VarOtF)
# R2[GLMM(c)] - conditional R2[GLMM] for full model
R2glmmC <- (VarF + sum(as.numeric(VarCorr(explmodPQLf)[c(2, 4), 1])))/(VarF +
    sum(as.numeric(VarCorr(explmodPQLf)[c(2, 4), 1])) + VarOtF)
# Raw unadjusted ICC[Population]
ICCrawPop <- as.numeric(VarCorr(explmodPQLr)[2, 1])/(sum(as.numeric(VarCorr(explmodPQLr)[c(2,
    4), 1])) + VarOtN)
# adjusted ICC[Population]
ICCadjPop <- as.numeric(VarCorr(explmodPQLf)[2, 1])/(sum(as.numeric(VarCorr(explmodPQLf)[c(2,
    4), 1])) + VarOtF)
# Raw unadjusted ICC[Container]
ICCrawCont <- as.numeric(VarCorr(explmodPQLr)[4, 1])/(sum(as.numeric(VarCorr(explmodPQLr)[c(2,
    4), 1])) + VarOtN)
# adjusted ICC[Container]
ICCadjCont <- as.numeric(VarCorr(explmodPQLf)[4, 1])/(sum(as.numeric(VarCorr(explmodPQLf)[c(2,
    4), 1])) + VarOtF)
# comparing the results
c(R2glmmM = R2glmmM, R2glmmC = R2glmmC, ICCrawPop = ICCrawPop, ICCadjPop = ICCadjPop,
    ICCrawCont = ICCrawCont, ICCadjCont = ICCadjCont)
```

```
##    R2glmmM    R2glmmC  ICCrawPop  ICCadjPop ICCrawCont ICCadjCont
## 0.78959865 0.94190914 0.04362017 0.27951061 0.23303547 0.44439394
```

(4.3) glmer exploration models

```
# Fit null model without fixed effects (but including all random effects)
explmodGLMERr <- glmer(Exploration ~ 1 + (1 | Population) + (1 | Container),
    family = Gamma(link = log), data = DataAll)
# Fit alternative model including fixed and all random effects
explmodGLMERf <- glmer(Exploration ~ Sex + Treatment + Habitat + (1 | Population) +
    (1 | Container), family = Gamma(link = log), data = DataAll)
# View model fits for both models
summary(explmodGLMERr)
```

```
## Generalized linear mixed model fit by maximum likelihood (Laplace
##   Approximation) [glmerMod]
##  Family: Gamma  ( log )
## Formula: Exploration ~ 1 + (1 | Population) + (1 | Container)
##    Data: DataAll
##
##      AIC      BIC   logLik deviance df.resid
##  11234.7  11254.2  -5613.4  11226.7      956
##
## Scaled residuals:
##     Min      1Q  Median      3Q     Max
## -1.0857 -0.8400 -0.4647  0.7478  2.2763
##
## Random effects:
##  Groups     Name        Variance Std.Dev.
##  Container  (Intercept) 0.32991  0.5744
##  Population (Intercept) 0.06202  0.2490
##  Residual               0.83788  0.9154
## Number of obs: 960, groups:  Container, 120; Population, 12
##
## Fixed effects:
##             Estimate Std. Error t value Pr(>|z|)
## (Intercept)   4.6995     0.1021   46.01   <2e-16 ***
## ---
## Signif. codes:  0 '***' 0.001 '**' 0.01 '*' 0.05 '.' 0.1 ' ' 1
```

```
summary(explmodGLMERf)
```

```
## Generalized linear mixed model fit by maximum likelihood (Laplace
##   Approximation) [glmerMod]
##  Family: Gamma  ( log )
## Formula: Exploration ~ Sex + Treatment + Habitat + (1 | Population) +
##     (1 | Container)
##    Data: DataAll
##
##      AIC      BIC   logLik deviance df.resid
##     8852     8886    -4419     8838      953
##
## Scaled residuals:
##     Min      1Q  Median      3Q     Max
## -2.7783 -0.4509 -0.0243  0.4693  5.5650
##
## Random effects:
```

```
##  Groups     Name        Variance Std.Dev.
##  Container  (Intercept) 0.06924  0.2631
##  Population (Intercept) 0.01896  0.1377
##  Residual               0.11713  0.3422
## Number of obs: 960, groups:  Container, 120; Population, 12
##
## Fixed effects:
##              Estimate Std. Error t value Pr(>|z|)
## (Intercept)   4.02695    0.12589   31.99   <2e-16 ***
## SexMale      -1.10733    0.09723  -11.39   <2e-16 ***
## TreatmentExp  2.00823    0.02091   96.06   <2e-16 ***
## HabitatWet   -0.56120    0.02088  -26.88   <2e-16 ***
## ---
## Signif. codes:  0 '***' 0.001 '**' 0.01 '*' 0.05 '.' 0.1 ' ' 1
##
## Correlation of Fixed Effects:
##             (Intr) SexMal TrtmnE
## SexMale     -0.386
## TreatmntExp -0.081 -0.002
## HabitatWet  -0.083  0.003 -0.019
```

```
# Calculation of the variance in fitted values
VarF <- var(as.vector(model.matrix(explmodGLMERf) %*% fixef(explmodGLMERf)))

# getting the observation-level variance Null model
nuN <- 1/attr(VarCorr(explmodGLMERr), "sc")^2  # note that glmer report 1/nu not nu as resiudal varianc
VarOdN <- 1/nuN  # the delta method
VarOlN <- log(1 + 1/nuN)  # log-normal approximation
VarOtN <- trigamma(nuN)  # trigamma function
# comparing the three
c(VarOdN = VarOdN, VarOlN = VarOlN, VarOtN = VarOtN)
```

```
##    VarOdN    VarOlN    VarOtN
## 0.8378761 0.6086106 1.2770627
```

```
# Full model
nuF <- 1/attr(VarCorr(explmodGLMERf), "sc")^2  # note that glmer report 1/nu not nu as resiudal varianc
VarOdF <- 1/nuF  # the delta method
VarOlF <- log(1 + 1/nuF)  # log-normal approximation
VarOtF <- trigamma(nuF)  # trigamma function
# comparing the three
c(VarOdF = VarOdF, VarOlF = VarOlF, VarOtF = VarOtF)
```

```
##    VarOdF    VarOlF    VarOtF
## 0.1171296 0.1107625 0.1242564
```

```
# R2[GLMM(m)] - marginal R2[GLMM]
R2glmmM <- VarF/(VarF + sum(as.numeric(VarCorr(explmodGLMERf))) + VarOtF)
# R2[GLMM(c)] - conditional R2[GLMM] for full model
R2glmmC <- (VarF + sum(as.numeric(VarCorr(explmodGLMERf))))/(VarF + sum(as.numeric(VarCorr(explmodGLMER
    VarOtF)
# Raw unadjusted ICC[Population]
ICCrawPop <- as.numeric(VarCorr(explmodGLMERr)$Population)/(sum(as.numeric(VarCorr(explmodGLMERr))) +
    VarOtN)
# adjusted ICC[Population]
ICCadjPop <- as.numeric(VarCorr(explmodGLMERf)$Population)/(sum(as.numeric(VarCorr(explmodGLMERf))) +
```

```
    VarOtF)
# Raw unadjusted ICC[Container]
ICCrawCont <- as.numeric(VarCorr(explmodGLMERr)$Container)/(sum(as.numeric(VarCorr(explmodGLMERr))) +
    VarOtN)
# adjusted ICC[Container]
ICCadjCont <- as.numeric(VarCorr(explmodGLMERf)$Container)/(sum(as.numeric(VarCorr(explmodGLMERf))) +
    VarOtF)
# comparing the results
c(R2glmmM = R2glmmM, R2glmmC = R2glmmC, ICCrawPop = ICCrawPop, ICCadjPop = ICCadjPop,
    ICCrawCont = ICCrawCont, ICCadjCont = ICCadjCont)
```

```
##    R2glmmM    R2glmmC  ICCrawPop  ICCadjPop ICCrawCont ICCadjCont
## 0.86782820 0.92269908 0.03715981 0.08923756 0.19767104 0.32591065
```

(5) Morph models: Binomial (binary) GLMMs with logit link

Below we use two founctions: `glmmabmb` and `glmer`. They produce similar results both for regression coefficents (fixed effects) and variance components (random effects).

(5.1) `glmmadmb` morph models

```
# Fit null model without fixed effects (but including all random effects)
morphmodADMBr <- glmmadmb(Colour ~ 1 + (1 | Population) + (1 | Container), family = "binomial",
    data = DataMale)
# Fit alternative model including fixed and all random effects
morphmodADMBf <- glmmadmb(Colour ~ Treatment + Habitat + (1 | Population) +
    (1 | Container), family = "binomial", data = DataMale)
# View model fits for both models
summary(morphmodADMBr)
```

```
##
## Call:
## glmmadmb(formula = Colour ~ 1 + (1 | Population) + (1 | Container),
##     data = DataMale, family = "binomial")
##
## AIC: 605.5
##
## Coefficients:
##             Estimate Std. Error z value Pr(>|z|)
## (Intercept)   -0.108      0.311   -0.35     0.73
##
## Number of observations: total=480, Population=12, Container=60
## Random effect variance(s):
## Group=Population
##             Variance StdDev
## (Intercept)    1.002  1.001
## Group=Container
##             Variance StdDev
## (Intercept)   0.1356 0.3683
##
##
## Log-likelihood: -299.759
```

```r
summary(morphmodADMBf)
```

```
##
## Call:
## glmmadmb(formula = Colour ~ Treatment + Habitat + (1 | Population) +
##     (1 | Container), data = DataMale, family = "binomial")
##
## AIC: 589.6
##
## Coefficients:
##              Estimate Std. Error z value Pr(>|z|)
## (Intercept)    -0.740      0.362   -2.04    0.041 *
## TreatmentExp    0.840      0.213    3.94  8.2e-05 ***
## HabitatWet      0.414      0.210    1.97    0.049 *
## ---
## Signif. codes:  0 '***' 0.001 '**' 0.01 '*' 0.05 '.' 0.1 ' ' 1
##
## Number of observations: total=480, Population=12, Container=60
## Random effect variance(s):
## Group=Population
##             Variance StdDev
## (Intercept)    1.111  1.054
## Group=Container
##             Variance StdDev
## (Intercept)   0.1857 0.4309
##
##
## Log-likelihood: -289.801
```

```r
# Calculation of the variance in fitted values
VarF <- var(as.vector(model.matrix(morphmodADMBf) %*% fixef(morphmodADMBf)))

# getting the (theortical) distribuiotn-specific and observation-level
# variance
VarDS <- pi^2/3
Vt <- VarCorr(morphmodADMBf)$Population[1] + VarCorr(morphmodADMBf)$Container[1]
pmean <- plogis(as.numeric(fixef(morphmodADMBr)) - 0.5 * Vt * tanh(as.numeric(fixef(morphmodADMBr)) *
    (1 + 2 * exp(-0.5 * Vt))/6))
VarOL <- 1/(pmean * (1 - pmean))
# VarOL2<-1/(mean(DataMale$Colour)*(1-mean(DataMale$Colour))) # the delta
# method comparing the two
c(VarDS = VarDS, VarOL = VarOL)
```

```
##    VarDS    VarOL
## 3.289868 4.007114
```

```r
# Theortical R2[GLMM(m)] - marginal R2[GLMM]
R2glmmM <- VarF/(VarF + sum(as.numeric(VarCorr(morphmodADMBf))) + VarDS)
# R2[GLMM(c)] - conditional R2[GLMM] for full model
R2glmmC <- (VarF + sum(as.numeric(VarCorr(morphmodADMBf))))/(VarF + sum(as.numeric(VarCorr(morphmodADMB
    VarDS)
# Raw unadjusted ICC[Population]
ICCrawPop <- VarCorr(morphmodADMBr)$Population[1]/(sum(as.numeric(VarCorr(morphmodADMBr))) +
    VarDS)
# adjusted ICC[Population]
```

```
ICCadjPop <- VarCorr(morphmodADMBf)$Population[1]/(sum(as.numeric(VarCorr(morphmodADMBf))) +
    VarDS)
# Raw unadjusted ICC[Container]
ICCrawCont <- VarCorr(morphmodADMBr)$Container[1]/(sum(as.numeric(VarCorr(morphmodADMBr))) +
    VarDS)
# adjusted ICC[Container]
ICCadjCont <- VarCorr(morphmodADMBf)$Container[1]/(sum(as.numeric(VarCorr(morphmodADMBf))) +
    VarDS)
# summarizing the results
Theory <- c(R2glmmM = R2glmmM, R2glmmC = R2glmmC, ICCrawPop = ICCrawPop, ICCadjPop = ICCadjPop,
    ICCrawCont = ICCrawCont, ICCadjCont = ICCadjCont)

# Observation-level R2[GLMM(m)] - marginal R2[GLMM]
R2glmmM <- VarF/(VarF + sum(as.numeric(VarCorr(morphmodADMBf))) + VarOL)
# R2[GLMM(c)] - conditional R2[GLMM] for full model
R2glmmC <- (VarF + sum(as.numeric(VarCorr(morphmodADMBf))))/(VarF + sum(as.numeric(VarCorr(morphmodADMB
    VarOL)
# Raw unadjusted ICC[Population]
ICCrawPop <- VarCorr(morphmodADMBr)$Population[1]/(sum(as.numeric(VarCorr(morphmodADMBr))) +
    VarOL)
# adjusted ICC[Population]
ICCadjPop <- VarCorr(morphmodADMBf)$Population[1]/(sum(as.numeric(VarCorr(morphmodADMBf))) +
    VarOL)
# Raw unadjusted ICC[Container]
ICCrawCont <- VarCorr(morphmodADMBr)$Container[1]/(sum(as.numeric(VarCorr(morphmodADMBr))) +
    VarOL)
# adjusted ICC[Container]
ICCadjCont <- VarCorr(morphmodADMBf)$Container[1]/(sum(as.numeric(VarCorr(morphmodADMBf))) +
    VarOL)
# summarizing the results
Obs <- c(R2glmmM = R2glmmM, R2glmmC = R2glmmC, ICCrawPop = ICCrawPop, ICCadjPop = ICCadjPop,
    ICCrawCont = ICCrawCont, ICCadjCont = ICCadjCont)
# comparing the results
rbind(Theory, Obs)
```

```
##           R2glmmM   R2glmmC ICCrawPop ICCadjPop ICCrawCont ICCadjCont
## Theory 0.04570632 0.3155430 0.2263823 0.2422797 0.03063298 0.04048096
## Obs    0.03977150 0.2745707 0.1948241 0.2095174 0.02636267 0.03500692
```

(5.2) glmer morph models

```
# Fit null model without fixed effects (but including all random effects)
morphmodGLMERr <- glmer(Colour ~ 1 + (1 | Population) + (1 | Container), family = binomial(link = logit
    data = DataMale)
# Fit alternative model including fixed and all random effects
morphmodGLMERf <- glmer(Colour ~ Treatment + Habitat + (1 | Population) + (1 |
    Container), family = binomial(link = logit), data = DataMale)
# View model fits for both models
summary(morphmodGLMERr)
```

```
## Generalized linear mixed model fit by maximum likelihood (Laplace
##   Approximation) [glmerMod]
##  Family: binomial  ( logit )
```

```
## Formula: Colour ~ 1 + (1 | Population) + (1 | Container)
##    Data: DataMale
##
##      AIC      BIC   logLik deviance df.resid
##    605.5    618.0   -299.8    599.5      477
##
## Scaled residuals:
##     Min      1Q  Median      3Q     Max
## -1.9949 -0.7198 -0.3566  0.6990  2.6388
##
## Random effects:
##  Groups     Name        Variance Std.Dev.
##  Container  (Intercept) 0.1355   0.3681
##  Population (Intercept) 1.0023   1.0012
## Number of obs: 480, groups:  Container, 60; Population, 12
##
## Fixed effects:
##             Estimate Std. Error z value Pr(>|z|)
## (Intercept)  -0.1083     0.3107  -0.348    0.727
```

```
summary(morphmodGLMERf)
```

```
## Generalized linear mixed model fit by maximum likelihood (Laplace
##   Approximation) [glmerMod]
##  Family: binomial  ( logit )
## Formula: Colour ~ Treatment + Habitat + (1 | Population) + (1 | Container)
##    Data: DataMale
##
##      AIC      BIC   logLik deviance df.resid
##    589.6    610.5   -289.8    579.6      475
##
## Scaled residuals:
##     Min      1Q  Median      3Q     Max
## -2.3250 -0.7388 -0.3001  0.7524  3.7796
##
## Random effects:
##  Groups     Name        Variance Std.Dev.
##  Container  (Intercept) 0.1855   0.4308
##  Population (Intercept) 1.1108   1.0540
## Number of obs: 480, groups:  Container, 60; Population, 12
##
## Fixed effects:
##              Estimate Std. Error z value Pr(>|z|)
## (Intercept)   -0.7399     0.3614  -2.047   0.0406 *
## TreatmentExp   0.8394     0.2117   3.966 7.31e-05 ***
## HabitatWet     0.4137     0.2087   1.982   0.0475 *
## ---
## Signif. codes:  0 '***' 0.001 '**' 0.01 '*' 0.05 '.' 0.1 ' ' 1
##
## Correlation of Fixed Effects:
##             (Intr) TrtmnE
## TreatmntExp -0.310
## HabitatWet  -0.303  0.041
```

```r
# Calculation of the variance in fitted values
VarF <- var(as.vector(model.matrix(morphmodGLMERf) %*% fixef(morphmodGLMERf)))

# getting the (theortical) distribuiotn-specific and observation-level
# variance
VarDS <- pi^2/3
Vt <- VarCorr(morphmodGLMERr)$Population + VarCorr(morphmodGLMERr)$Container
pmean <- plogis(as.numeric(fixef(morphmodGLMERr)) - 0.5 * Vt * tanh(as.numeric(fixef(morphmodGLMERr)) *
    (1 + 2 * exp(-0.5 * Vt))/6))
VarOL <- 1/(pmean * (1 - pmean))
# VarOL2<-1/(mean(DataMale$Colour)*(1-mean(DataMale$Colour))) # the delta
# method comparing the two
c(VarDS = VarDS, VarOL = VarOL)
```

```
##    VarDS    VarOL
## 3.289868 4.007469
```

```r
# Theoritical R2[GLMM(m)] - marginal R2[GLMM]
R2glmmM <- VarF/(VarF + sum(as.numeric(VarCorr(morphmodGLMERf))) + VarDS)
# R2[GLMM(c)] - conditional R2[GLMM] for full model
R2glmmC <- (VarF + sum(as.numeric(VarCorr(morphmodGLMERf))))/(VarF + sum(as.numeric(VarCorr(morphmodGLM
    VarDS)
# Raw unadjusted ICC[Population]
ICCrawPop <- as.numeric(VarCorr(morphmodGLMERr)$Population)/(sum(as.numeric(VarCorr(morphmodGLMERr))) +
    VarDS)
# adjusted ICC[Population]
ICCadjPop <- as.numeric(VarCorr(morphmodGLMERf)$Population)/(sum(as.numeric(VarCorr(morphmodGLMERf))) +
    VarDS)
# Raw unadjusted ICC[Container]
ICCrawCont <- as.numeric(VarCorr(morphmodGLMERr)$Container)/(sum(as.numeric(VarCorr(morphmodGLMERr))) +
    VarDS)
# adjusted ICC[Container]
ICCadjCont <- as.numeric(VarCorr(morphmodGLMERf)$Container)/(sum(as.numeric(VarCorr(morphmodGLMERf))) +
    VarDS)
# summarizing the results
Theory <- c(R2glmmM = R2glmmM, R2glmmC = R2glmmC, ICCrawPop = ICCrawPop, ICCadjPop = ICCadjPop,
    ICCrawCont = ICCrawCont, ICCadjCont = ICCadjCont)

# Observation-level R2[GLMM(m)] - marginal R2[GLMM]
R2glmmM <- VarF/(VarF + sum(as.numeric(VarCorr(morphmodGLMERf))) + VarOL)
# R2[GLMM(c)] - conditional R2[GLMM] for full model
R2glmmC <- (VarF + sum(as.numeric(VarCorr(morphmodGLMERf))))/(VarF + sum(as.numeric(VarCorr(morphmodGLM
    VarOL)
# Raw unadjusted ICC[Population]
ICCrawPop <- as.numeric(VarCorr(morphmodGLMERr)$Population)/(sum(as.numeric(VarCorr(morphmodGLMERr))) +
    VarOL)
# adjusted ICC[Population]
ICCadjPop <- as.numeric(VarCorr(morphmodGLMERf)$Population)/(sum(as.numeric(VarCorr(morphmodGLMERf))) +
    VarOL)
# Raw unadjusted ICC[Container]
ICCrawCont <- as.numeric(VarCorr(morphmodGLMERr)$Container)/(sum(as.numeric(VarCorr(morphmodGLMERr))) +
    VarOL)
# adjusted ICC[Container]
ICCadjCont <- as.numeric(VarCorr(morphmodGLMERf)$Container)/(sum(as.numeric(VarCorr(morphmodGLMERf))) +
```

```
    VarOL)
# summarizing the results
Obs <- c(R2glmmM = R2glmmM, R2glmmC = R2glmmC, ICCrawPop = ICCrawPop, ICCadjPop = ICCadjPop,
    ICCrawCont = ICCrawCont, ICCadjCont = ICCadjCont)
# comparing the results
rbind(Theory, Obs)
```

```
##           R2glmmM   R2glmmC ICCrawPop ICCadjPop ICCrawCont ICCadjCont
## Theory 0.04565558 0.3154186 0.2263791 0.2422109 0.03059611 0.04045751
## Obs    0.03972385 0.2744383 0.1948064 0.2094402 0.02632893 0.03498370
```

(6) *Extra* parasite models: Tweedie (Compound Poisson) GLMMs with log link

R^2_{GLMM} and ICC_{GLMM} obtained from the `cpglmm` models were very similar to those from the `glmmabmb` models and the `glmer.nb` models above.

```
library(cplm)
```

(6.1) cpglmm parasite models

```
# Fit null model without fixed effects (but including all random effects)
parmodCPr <- cpglmm(Parasite ~ 1 + (1 | Population) + (1 | Container), data = DataAll)
# Fit alternative model including fixed and all random effects
parmodCPf <- cpglmm(Parasite ~ Sex + Treatment + Habitat + (1 | Population) +
    (1 | Container), data = DataAll)
# View model fits for both models
summary(parmodCPr)
```

```
## Compound Poisson linear mixed model fit by the Laplace approximation
## Formula: Parasite ~ 1 + (1 | Population) + (1 | Container)
##    Data: DataAll
##   AIC  BIC logLik deviance
##  4560 4579  -2276     4552
## Random effects:
##  Groups     Name        Variance Std.Dev.
##  Container  (Intercept) 1.93071  1.38950
##  Population (Intercept) 0.37748  0.61439
##  Residual               2.28393  1.51127
## Number of obs: 960, groups: Container, 120; Population, 12
##
## Fixed effects:
##             Estimate Std. Error t value
## (Intercept)   0.7572     0.2223   3.406
##
## Estimated dispersion parameter: 2.2839
## Estimated index parameter: 1.3685
```

```
summary(parmodCPf)
```

```
## Compound Poisson linear mixed model fit by the Laplace approximation
## Formula: Parasite ~ Sex + Treatment + Habitat + (1 | Population) + (1 |      Container)
##    Data: DataAll
##   AIC  BIC logLik deviance
```

```
##  4045 4079  -2015     4031
## Random effects:
##  Groups     Name        Variance Std.Dev.
##  Container  (Intercept) 0.60049  0.77492
##  Population (Intercept) 0.54026  0.73503
##  Residual               1.70038  1.30399
## Number of obs: 960, groups: Container, 120; Population, 12
##
## Fixed effects:
##               Estimate Std. Error t value
## (Intercept)    1.72071    0.23916   7.195
## SexMale       -2.21471    0.16144 -13.719
## TreatmentExp -0.76009    0.04432 -17.150
## HabitatWet     0.74759    0.04426  16.892
##
## Estimated dispersion parameter: 1.7004
## Estimated index parameter: 1.213
```

```
# Calculation of the variance in fitted values
VarF <- var(as.vector(model.matrix(parmodCPf) %*% fixef(parmodCPf)))

# getting the observation-level variance Null model
phiN <- parmodCPr@phi  # the dispersion parameter
pN <- parmodCPr@p  # the index parameter
mu <- exp(fixef(parmodCPr) + 0.5 * (VarCorr(parmodCPr)$Population[1] + VarCorr(parmodCPr)$Container[1]))
# mu2 <- mean(DataAll$Parasite) # the mean on the data scale (lambda)
VarOdN <- phiN * mu^(pN - 2)  # the delta method
VarOlN <- log(1 + (phiN * mu^(pN - 2)))  # log-normal approximation

# comparing the three
c(VarOdN = VarOdN, VarOlN = VarOlN)
```

```
## VarOdN.(Intercept) VarOlN.(Intercept)
##          0.6831562          0.5206707
```

```
# Full model
phiF <- parmodCPf@phi  # the dispersion parameter
pF <- parmodCPf@p  # the index parameter
VarOdF <- phiF * mu^(pF - 2)  # the delta method
VarOlF <- log(1 + (phiF * mu^(pF - 2)))  # log-normal approximation
# comparing the three
c(VarOdF = VarOdF, VarOlF = VarOlF)
```

```
## VarOdF.(Intercept) VarOlF.(Intercept)
##          0.3778427          0.3205190
```

```
# R2[GLMM(m)] - marginal R2[GLMM]; using the delta method observation-level
# variance
R2glmmM <- VarF/(VarF + sum(as.numeric(VarCorr(parmodCPf))) + VarOdF)
# R2[GLMM(c)] - conditional R2[GLMM] for full model
R2glmmC <- (VarF + sum(as.numeric(VarCorr(parmodCPf))))/(VarF + sum(as.numeric(VarCorr(parmodCPf))) +
    VarOdF)
# Raw unadjusted ICC[Population]
ICCrawPop <- VarCorr(parmodCPr)$Population[1]/(sum(as.numeric(VarCorr(parmodCPr))) +
    VarOdN)
# adjusted ICC[Population]
```

```r
ICCadjPop <- VarCorr(parmodCPf)$Population[1]/(sum(as.numeric(VarCorr(parmodCPf))) +
    VarOdF)
# Raw unadjusted ICC[Container]
ICCrawCont <- VarCorr(parmodCPr)$Container[1]/(sum(as.numeric(VarCorr(parmodCPr))) +
    VarOdN)
# adjusted ICC[Container]
ICCadjCont <- VarCorr(parmodCPf)$Container[1]/(sum(as.numeric(VarCorr(parmodCPf))) +
    VarOdF)
# comparing the results
c(R2glmmM = R2glmmM, R2glmmC = R2glmmC, ICCrawPop = ICCrawPop, ICCadjPop = ICCadjPop,
    ICCrawCont = ICCrawCont, ICCadjCont = ICCadjCont)
```

```
##    R2glmmM.(Intercept)    R2glmmC.(Intercept)  ICCrawPop.(Intercept)
##              0.4989050              0.8753228              0.1261894
##  ICCadjPop.(Intercept) ICCrawCont.(Intercept) ICCadjCont.(Intercept)
##              0.3557646              0.6454326              0.3954258
```

This is the end (Thank you)

Please contact us (Shinichi Nakagawa; `s.nakagawa@unsw.edu.au` or Holger Schielzeth; `holger.schielzeth@uni-jena.de`) if you find mistakes and bugs.
